# Robust *In Vitro* Pharmacology of Tmod, a Synthetic Dual-Signal Integrator for Cancer Cell Therapy

**DOI:** 10.3389/fimmu.2022.826747

**Published:** 2022-03-10

**Authors:** Diane Manry, Kristian Bolanos, Breanna DiAndreth, Jee-Young Mock, Alexander Kamb

**Affiliations:** A2 Biotherapeutics, Inc., Agoura Hills, CA, United States

**Keywords:** NOT gate logic, competitive antagonist, receptor pharmacology, tumor deletion, CAR-T, LILR1

## Abstract

Progress toward improved solid-tumor treatment has long been hindered by the lack of truly tumor-specific targets. We have developed an approach to T cell therapy based on a dual-receptor system called Tmod™ that addresses this problem. The Tmod system exploits one of the few common genetic differences between tumor and normal cells: loss of heterozygosity (LOH). It utilizes the basic mechanistic logic that evolved in early vertebrates to mediate self vs. non-self discrimination, where an activation stimulus is blocked by self-ligands. Tmod constructs employ a chimeric antigen receptor (CAR) or T cell receptor (TCR) as activator component and a modified LIR-1 inhibitory receptor (blocker) to achieve high selectivity based on expression of the blocker antigen (Ag). Here we explore the *in vitro* pharmacology of a blocker directed at the HLA-A*02 Ag paired with either a mesothelin CAR or an HLA-A*11-restricted KRAS peptide TCR. While more sensitive to receptor expression changes on effector cells, we show that Tmod response is well-buffered against variations in Ag levels on target cells. In addition, the data reveal at least two distinguishable pharmacologic mechanisms of Tmod blocker function: (1) reducing activator sensitivity and (2) decreasing activation magnitude.

## 1 Introduction

The fundamental challenge of cancer therapy is therapeutic discrimination between tumor and normal cells. Two decades after the publication of the draft human genome sequence, we now have definitive evidence for the type and frequency of genetic changes in cancer cells (1). There are numerous instances of nucleotide substitutions, microdeletions, and amplifications that result in quantitative or qualitative gain of Ag expression in the tumor. Deletions that result in loss of Ag expression constitute an even larger portion of the average cancer genome. Though homozygous deletions are rare, loss of heterozygosity (LOH) is extremely common, affecting ~20% of genes in a typical cancer (2). LOH confers an opportunity to distinguish tumor from normal cells by targeting these genetic losses with a cellular NOT logic gate designed to mediate the attack of engineered immune cells on cancer cells that lack expression of a specific Ag, while sparing normal cells that possess the Ag (3).

We and others have devised various logic mechanisms for signal integration (4–9). Our system, Tmod, has two components: an activator based on a CAR or TCR and a blocker based on the LIR-1 inhibitory receptor that is predominantly expressed in monocytes (10). As one example, we are developing a Tmod therapeutic that targets mesothelin (MSLN), which is expressed on many solid tumors, and employs a blocker directed at the HLA-A*02 Ag expressed ubiquitously on tissues of A*02(+) individuals (11). By selecting patients who carry: (i) germline heterozygous A*02 alleles; and, (ii) clonal A*02 LOH in their tumor, we intend to specifically target their tumors with Tmod-engineered T cells.

The Tmod system has remarkable properties. It is modular and can accommodate multiple target and receptor types (5, 12). It is reversible and can cycle between ON and OFF states. It directs engineered T cells to kill tumor cells with great specificity, even in co-culture with a 10-fold excess of normal cells. Finally, it can achieve absolute potency and selectivity levels that rival the adaptive immune response (11, 13). Here we explore the pharmacology of Tmod *in vitro* by systematic variation of Ag and/or receptor inputs. Despite the multi-component system of cells, Ags and receptors, quantitative experiments demonstrate a smooth, buffered dose-response over a range of inputs. These findings provide confidence in the reliability of the fundamental mechanism and its potential utility for cancer therapy.

## 2 Methods

### 2.1 Cell Lines and Cell Culture

The Jurkat cell line containing an NFAT luciferase reporter was obtained from BPS Bioscience. For MSLN Tmod and KRAS Tmod two-dimensional Ag titration, Jurkat cells were engineered by transduction with MSLN CAR + A*02 blocker lentivirus (custom lentivirus, Lentigen) and KRAS TCR lentivirus + A*02 blocker lentivirus (custom lentivirus, Alstem), respectively, at an MOI of 5, followed by enrichment using Anti-PE and/or Anti-APC MicroBeads (MACS Miltenyi Biotec). Jurkat cells were grown in RPMI media with 10% heat-inactivated FBS, 1X Pen/Strep, and 400 ug/ml Geneticin. HeLa and T2 cell lines were obtained from ATCC and grown according to ATCC guidelines. The Hela MSLN knockout (KO) cell line was generated using CRISPR-mediated KO of the MSLN gene. Single cell clones were isolated and successful KOs were validated using MSLN antibody staining, flow cytometry, and functional validation with MSLN CAR. One such clone was used for relevant experiments in this study. For primary T cell experiments, target cells were transduced with lentivirus to introduce Renilla Luciferase-driven GFP expression (Biosettia), then sorted using a FACSMelody Sorter (BD). T2-A*11 cells were generated from a T2 HLA-A, -B, -C KO cell line (CRISPR-mediated KO) that was then transduced with HLA-A*11 lentivirus (custom lentivirus, Alstem). HLA-A*11-positive T2 cells were sorted using a FACSMelody Cell Sorter (BD).

### 2.2 Plasmid Constructs

The MSLN CAR construct consists of a novel anti-MSLN scFv ligand-binding domain (LBD) fused to a CD8α hinge, CD28 transmembrane region, and CD28, 4-1BB and CD3z signaling domains (**Table 1**; 11). The A*02 blocker was described in an earlier study (5) and consists of an anti-A*02 scFv LBD fused to the hinge, transmembrane region, and intracellular domain of leukocyte immunoglobulin-like receptor subfamily B member 1, LILRB1 (LIR-1). The KRAS TCR is the same as previously described (14). Constructs were generated using Golden Gate cloning and inserted downstream of a human EF1α promoter in a lentiviral expression plasmid.

**Table 1:** Constructs used in experiments.

Target	Format	Construct details
**MSLN**	**scFv Gen-3 CAR**	**CD8 H, CD28 TM, CD28 ICD, 4-1BB ICD, CD3z ICD**
HLA-A*11/KRAS_8-17_ peptide	**Chimeric TCR**	**Murine constant and human variable TCRα/β segments**
HLA-A*02	**scFv blocker**	**LIR-1 H, LIR-1 TM, LIR-1 ICD**

Two activators, a CAR directed at MSLN and a TCR directed at A*11/KRAS peptide complex, and a single blocker directed at HLA-A*02 were used. See Methods for details. H, hinge; TM, transmembrane domain; ICD, intracellular domains.

### 2.3 mRNA Synthesis

Custom MSLN mRNA was obtained from TriLink. HLA-A*02 mRNA was produced by Aldevron. MSLN CAR, KRAS TCR, and A*02 blocker mRNA was generated using the HiScribe™ T7 Quick High Yield RNA Synthesis Kit (New England BioLabs). In brief, activator or blocker DNA templates were synthesized *via* PCR from lentiviral expression plasmids. PCR products were used as templates for *in vitro* transcription using the RNA synthesis kit, followed by cleanup using the Monarch^®^ RNA Cleanup Kit (New England BioLabs).

### 2.4 Jurkat Cell Transfection

For receptor titration experiments, Jurkat cells were transfected with the indicated amounts of activator and/or blocker mRNA per 1.5e6 cells using the 100 ul Neon electroporation system (Thermo Fisher Scientific) according to the manufacturer’s instructions, and using the settings: 1500 V, 3 pulses, 10 ms. Cells were then recovered in RPMI media with 20% heat-inactivated FBS and 0.1% Pen/Strep for 18-24 hours prior to co-culture.

### 2.5 Target Cell Transfection and Peptide Loading

Target cells were transfected with mRNA using the Lonza 4D-Nucleofector system according to the manufacturer’s instructions. The following optimal transfection conditions were identified for HeLa and T2 cell lines: Lonza SE buffer and program CN-114 (HeLa), Lonza SF buffer and program CA-148 (T2). Activator (A-) or blocker (B-) Ag mRNA was serially diluted 2- or 3-fold in cell-line-appropriate buffer before combining with a constant amount of the second mRNA (B- or A-Ag, respectively) and cells suspended in buffer. Transfection was performed in either 100 ul cuvettes or 16-well strips (Lonza). Transfected cells were recovered in cell-line specific media supplemented with 20% FBS and 0.1% Pen/Strep. Modified NY-ESO-1 peptide (SLLMWITQV) and mutant KRAS peptide (VVVGAVGVGK) were synthesized by Genscript. For peptide-loading, T2-A*11 cells were first transfected with mRNA, then recovered in media containing the indicated peptide concentration, resulting in exogenous KRAS and NY-ESO-1 peptide loading.

### 2.6 Flow Cytometry, Ag Staining and Quantification

MSLN CAR expression was determined using biotinylated human mesothelin (296-580) protein (ACROBiosystems, MSN-H82E9) conjugated to streptavidin-PE (Invitrogen, S866). A*02 blocker expression was measured using biotinylated HLA-A*02 loaded with irrelevant peptide (see 15, for details) conjugated to streptavidin-APC (Invitrogen, S868). KRAS TCR was stained using PE-labeled anti-mouse TCR β chain antibody (Biolegend, 109208). Activator receptor expression levels were quantified by taking the geometric mean fluorescence intensity (MFI), subtracting the background MFI (MFI when 0 ng receptor was transfected), then dividing by the lowest MFI (MFI from the assay with the lowest amount of mRNA, i.e. 1250 or 556 ng). For target cell Ags, cell-surface Ag quantification was performed using QIFIKIT according to the manufacturer’s instructions (Agilent Dako). In brief, target cells were first stained with mouse primary antibody against the Ag of interest, then stained with a secondary antibody in parallel with calibration beads loaded with a pre-determined amount of mouse monoclonal antibody. After flow cytometry of the beads and cell samples and determining the MFI, the known antibody molecule numbers from the calibration beads were used to fit a standard curve, which was then used to extrapolate molecule numbers for the cell samples based on their MFIs. The primary antibodies used for target Ags were as follows: MSLN, anti-hMSLN (R&D Systems, MAB32653); A*02, anti-human HLA-A*02 (BD Biosciences, 551230); and A*11, anti-human HLA-A, B, C (Invitrogen, MA511723). Jurkat, primary T, and target cells were stained 18-24 hours post-transfection for 60 min at 4° in PBS with 1% BSA, then run on a flow cytometer (BD FACSCanto II).

### 2.7 Jurkat-NFAT-Luciferase Activation Assay

Jurkat cells and target cells (HeLa, HeLa MSLN KO, T2-A*11) were transfected with the indicated receptor mRNA and target Ag mRNA, respectively, then allowed to recover in appropriate media (see above). Immediately after transfection, target cells were seeded in Corning^®^ 384-well Low Flange White Flat Bottom Polystyrene TC-treated Microplates at a density of 10-12,000 cells per 15 ul. 18-24 hours after transfection and target cell plating, Jurkat cells were counted and resuspended in RPMI media supplemented with 10% heat-inactivated FBS and 0.1% Pen/Strep at a density of 10-12,000 cells per 15 ul. 15 ul of the Jurkat cell suspension was then added to the previously plated target cells and incubated for 6 hours. Jurkat-NFAT luciferase luminescence following co-culture was measured using the ONE-Step Luciferase Assay System (BPS Bioscience). Each assay was performed in technical duplicates.

### 2.8 Primary T Culture, Expansion, Transduction, and Transfection

Human PBMCs from two donors were purchased from Stemcell Technologies. Frozen PBMCs were thawed in a 37°C water bath then plated in X-VIVO™ media (Lonza) supplemented with 5% human serum for 4 hours. Cells were then filtered and plated at a density of 1e6 cells/ml in X-VIVO media supplemented with 5% human serum, then stimulated with 1:100 TransAct™ (Miltenyi). The next day, cells were transduced with lentivirus at an MOI of 5. On the following day, transduced cells were supplemented with extra media with 5% human serum and 300 IU/ml IL-2. 5-7 days later, cells were moved to a 24-well G-Rex plate (Wilson Wolf) in LymphoONE media (Takara Bio) supplemented with 1% human serum and 300 IU/ml IL-2. 300 IU/ml IL-2 was added to culture every 2-3 days, and LymphoONE media was changed every 7 days. Two weeks after the initial thawing process, primary T cells were cryofrozen in appropriate aliquots. Prior to each experiment, one aliquot of primary T cells from each donor was thawed in a 37°C water bath and allowed to recover for 1-3 days in a 24-well G-Rex plate in X-VIVO media supplemented with 5% human serum and 300 IU/ml IL-2. For target Ag titration experiments, primary T cells transduced with MSLN CAR and A*02 Blocker were used. For receptor titration experiments, untransduced primary T cells were transfected with the indicated amounts of activator and blocker mRNA per 1e6 cells using the 100 ul Neon electroporation system (Thermo Fisher Scientific) according to the manufacturer’s instructions, and using the settings: 1700 V, 1 pulses, 20 ms. Transfected primary T cells were allowed to recover overnight in X-VIVO media supplemented with 5% human serum and 300 IU/ml IL-2 prior to being used in cytotoxicity assays (see *Methods 2.9*).

### 2.9 Primary T Cell *In Vitro* Cytotoxicity Assay

Transduced or transfected primary T cells were prepared (*Methods 2.8*) and HeLa MSLN KO Renilla luciferase-GFP targets were transfected as described earlier (*Methods 2.5*) 18-24 hours prior to co-culture. For primary T cytotoxicity assays, target Ag mRNA was diluted 3-fold instead of 2-fold. Immediately after transfection, target cells were seeded in Corning^®^ 384-well Low Flange Black Clear Bottom Polystyrene TC-treated Microplates at a density of 2,000 cells per 30 ul in LymphoONE media supplemented with 1% human serum. 18-24 hours after transfection and target cell plating, primary T cells were counted and resuspended in LymphoONE media supplemented with 1% human serum at a density of 2,000 cells per 30 ul. 30 ul of the primary T cell suspension was then added to the previously plated target cells. GFP and transmitted light images were obtained for all wells every 2 hours for 24 hours using ImageXpress Micro Confocal (IXM, Molecular Devices). 24 hours after co-culture, 10 ul supernatant was taken from wells and IFNγ production was detected using BD™ Cytometric Bead Array Human IFN-γ Flex Set (BD Biosciences). Well images obtained from IXM were analyzed using MetaXpress Image Acquisition and Analysis Software (Molecular Devices) to determine the total GFP area per well. The total GFP area in each well at a given timepoint was normalized to total GFP area for that well at time 0, giving a measure of relative target cell growth (Target cell growth). Percent killing for each well at a given timepoint was calculated relative to untransfected (UTF) or untransduced (UTD) primary T cell control wells as follows:


$$\%Killing=\frac{Average\;(Target\;cell\;growth_{UTF})-Target\;cell\;growth_{well\;of\;interest}}{Average\;(Target\;cell\;growth_{UTF})}*100$$

As each replicate was performed in triplicate, three percent-killing values were averaged to obtain the percent killing and standard deviation for each point on the dose-response curves. Four parameter non-linear regression analysis was performed as described below (*Methods 2.10*).

### 2.10 Calculations and Statistical Analysis

GraphPad Prism was used for all statistical analyses. All data are shown as the mean ± standard deviation (SD) unless otherwise stated. Statistical significance was determined using an ordinary one-way ANOVA followed by a Tukey’s multiple comparisons test. Significant differences are indicated in figures with asterisk(s). Two replicates were performed per experiment, with each replicate done in duplicate (Jurkat cell assays) or triplicate (primary T cell assays). Peptide and mRNA titration dose-response curves were fit to each replicate using a four-parameter non-linear regression analysis, and EC50, Emax, Emin, IC50, Imax, and Imin were calculated from the curves. Other calculations were performed using the equations below. Where fold-change is indicated, all values were divided by the lowest value in order to determine fold-change differences amongst them. Fold-changes are the average of two experimental replicates.


$$EC50\;shift=\frac{EC50\;(blocked)}{EC50}$$

$$Emax\;shift=\frac{Emax-Emin}{Emax\;(blocked)-Emin\;(blocked)}$$

## 3 Results

### 3.1 Experimental Design

**Figure 1:**
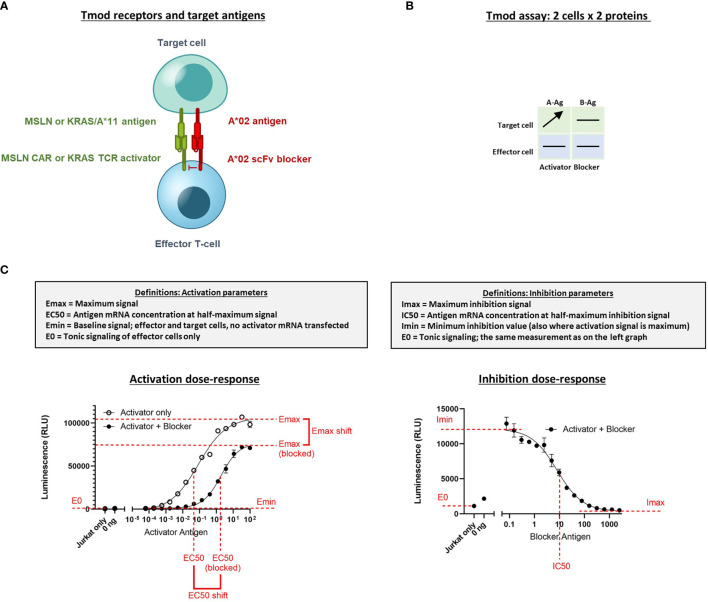
Diagram of Tmod system and assays to measure quantitative response in Jurkat cells. **(A)** Receptor components consist of an activator receptor (CAR or TCR) and a blocker receptor. The illustration shows a CAR, but the activator can also be a TCR. **(B)** The 2x2 box shows an example of a simple diagram that summarizes how the receptors/Ags are varied in the experiments described. Horizontal lines indicate constant protein level in experiment; arrow indicates titrated protein. In this example the A-Ag is titrated and the other molecules are held constant. A-Ag, activator Ag; B-Ag, blocker Ag. **(C)** Graphical illustration and definition of key parameters derived from Jurkat cell NFAT-luciferase reporter assays. Curves are example dose-response graphs.

We explored the behavior of two activator classes: (i) a CAR directed at MSLN (11); and, (ii) a TCR directed at a mutant KRAS peptide in complex with A*11 (14). These activators were paired with an A*02-directed peptide-independent blocker based on LIR-1 domains fused to an scFv derived from the PA2.1 monoclonal antibody (mAb; **Table 1**; **Figure 1A**) (5, 16). The goal was to generate two-dimensional titrations of both the Ags (A-Ag and B-Ag) and the receptors (activators and blocker) in Jurkat and primary T cells. The Jurkat NFAT-luciferase-reporter cell line provided a convenient quantitative measure of activation (17). When exploring effects of Ag levels, we sought to remove one source of variation inherent to transiently-transfected effector receptor levels. Therefore, we generated stable Tmod-engineered cells by transducing Jurkat and primary T cells with lentiviral vectors (see *Methods*).

The titration for Ag (MSLN, KRAS-pMHC, and A*02) and receptors (MSLN CAR, KRAS TCR, and A*02 blocker) was controlled either using transfected synthetic mRNA or *via* peptide-loading to span a wide range of Ag inputs (**Table 2**). The signal:noise properties of these assays considerably restricted the range over which different activator:blocker receptor ratios could be tested. Where possible, Ag surface expression was quantified (see *Methods*) and converted from mRNA (ng) to molecules/cell. Number of molecules/cell was used in place of mRNA amount when applicable. Because of the complexity of the experiment, we created a 2x2 diagram to illustrate the conditions of each experiment; i.e., which mRNA/proteins were varied in which cell types (**Figure 1B**). In most of the two-dimensional titrations, key activation and inhibition parameters were measured using a 14- or 16-point titration in one dimension, and a series of fixed concentrations in the second dimension (**Figure 1B**). These titrations produced data that could be fit using four-parameter non-linear regression, resulting in values of maximal response (Emax-Emin, or span of activation), half-maximal response (EC50, or activator sensitivity), etc., which could be analyzed further to draw conclusions about the relative effects under different conditions of Ag input (**Figure 1C**). As observed previously, maximal activation (Emax) was a direct function of the level of activator receptor expressed (15) (**Supplementary Figures 5C, 6C**). We inferred blocker activity from its inhibitory effect on the activator dose-response curve, as well as from IC50 (blocker sensitivity) and Imin-Imax (span of blocking). When comparing the effect of varying activator:blocker receptor ratios, we also calculated EC50 and Emax “shifts” that represent the fold-changes induced by presence of the blocker receptor.

**Table 2:** Range of inputs explored in different experiments.

Tmod Act	Titration	Dose-response assay type	A-Ag range (molecules/cell)	B-Ag range (molecules/cell)	Act range (fold)	Blk range (fold)
**MSLN**	**Ags**	Activation	0 – 1,000,000	0 –1,000,000	Constant	Constant
		Inhibition	3,000 – 330,000	0 – 1,000,000	Constant	Constant
	**Receptors**	Activation	0 – 1,000,000	Constant (3,000)	3.5x	Constant
		Inhibition	Constant (50,000)	0 – 625,000	3.5x	Constant
**KRAS**	**Ags**	Activation	0 – 80,000	0 – 200,000	Constant	Constant
		Inhibition	20,000 – 80,000	0 – 223,000	Constant	Constant
	**Receptors**	Activation	0 – 121,000	Constant (10,000)	14x	Constant
		Inhibition	Constant (20,000)	0 – 250,000	14x	Constant

Values are estimated from surface expression measurements and standard curves. For receptor titration experiments with MSLN Tmod, wildtype [i.e., MSLN(+)] Hela cells were used that express ~50,000 MSLN molecules/cell (see **Supplementary Figure 5B**). For KRAS Tmod experiments, T2 cells that stably express A*11 were used, and 50 μM NY-ESO-1 peptide was added to stabilize transfected A*02 expression on the cell surface (see text).

### 3.2 Tmod Ag Titration

#### 3.2.1 MSLN Tmod

In human tissues, surface Ag expression levels can vary dramatically. Because the utility of the Tmod system lies in its ability to distinguish tumor from normal tissues, recognizing varying amounts of A- and B-Ags is crucial for proper function. It is possible, for example, that a normal tissue with very high A-Ag and low B-Ag would not be recognized by Tmod effector cells as “normal”, resulting in on-target, off-tumor killing due to poor blocking. Therefore, we explored the effects of varying A- and B-Ag levels on Tmod activity.

**Figure 2:**
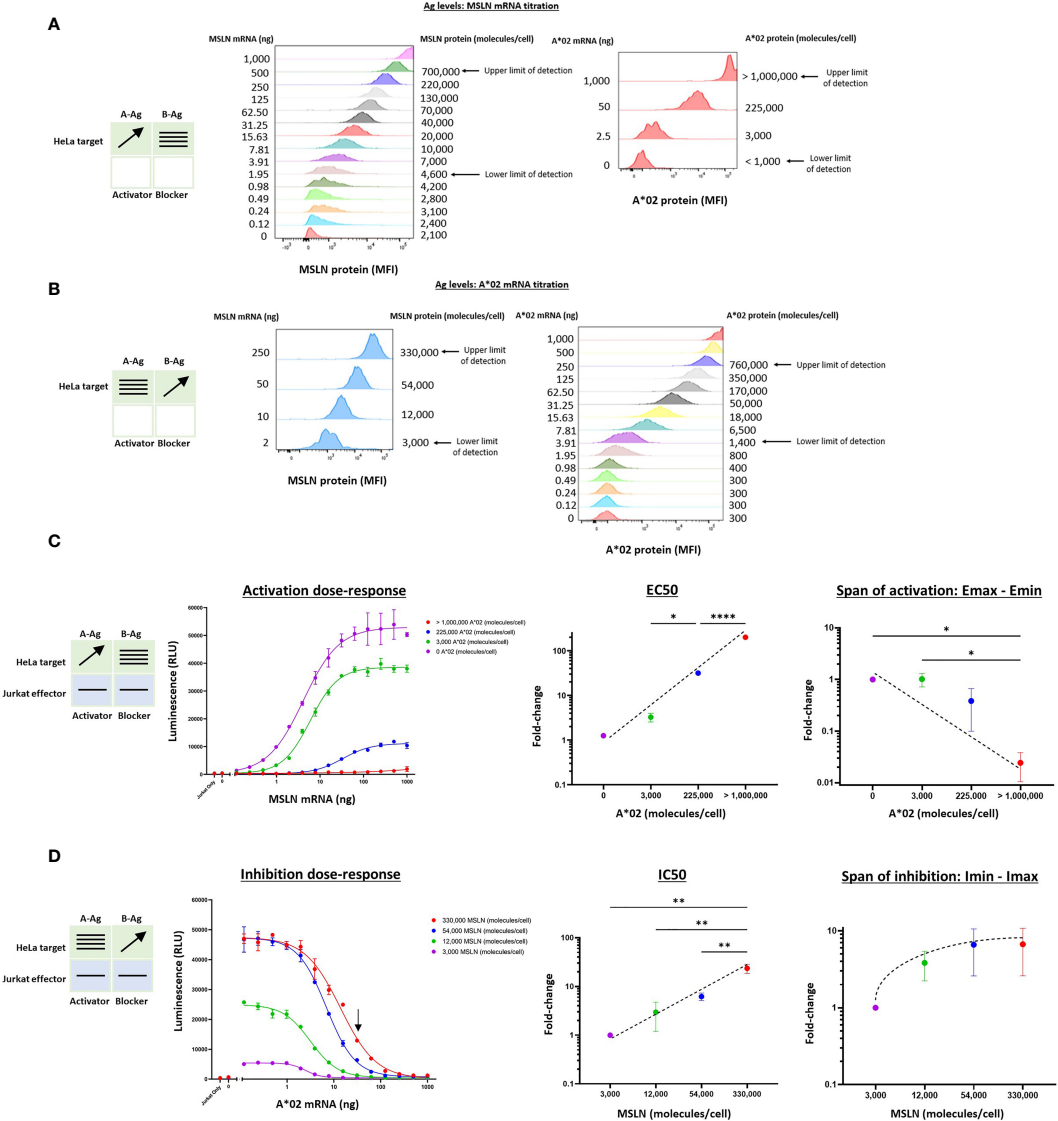
Two-dimensional target Ag (MSLN and A*02) mRNA titration in HeLa [A*02(-)MSLN(-)] target cells. Target cells were a HeLa variant with MSLN knocked out (see *Methods*). **(A)** MSLN (A-Ag) titration and surface expression visualized by flow cytometry. **(B)** A*02 (B-Ag) titration. The lower and upper limits of detection are shown. These define the observable boundaries of Ag inputs explored in the assay. See *Methods* for staining reagents, and **Supplementary Figure 1** for standard curves that relate mRNA level to molecules/cell. **(C)** Dose-response of MSLN and A*02 target Ag variation with constant activator/blocker ratio on effector cells. MSLN A-Ag titrated with varying A*02 B-Ag levels held constant (EC50 and Emax vs B-Ag levels). For the red curve (>1,000,000 A*02 molecules/cell), the EC50 was estimated to be the maximum MSLN Ag tested, since the curve did not increase or plateau and curve-fitting was inaccurate. Fold-change is calculated by normalizing to the lowest B-Ag value. **(D)** A*02 B-Ag titrated with varying MSLN A-Ag levels held constant (IC50 and Imax vs. A-Ag levels). Fold-change is calculated by normalizing to the lowest A-Ag value. Dashed lines are meant to convey trends and are not fit mathematically. *p<0.05, **p < 0.01, ***p < 0.001, ****p < 0.0001, n = 2.

We began by measuring the effects of target cells with varying MSLN and A*02 Ag levels on the activity of Jurkat cells stably expressing MSLN CAR and A*02 blocker (MSLN Tmod cells). As stimulus, we used a HeLa cell variant with the MSLN gene inactivated (HeLa MSLN KO, see *Methods*). Because HeLa cells lack A*02, we could titrate both the A-Ag (MSLN) and the B-Ag (A*02) in a target cell null for both. Variation of mRNA levels produced surface quantities of the two Ags that spanned a broad range of mean molecules/cell in largely uniform lognormal distributions (**Figures 2A, B**). By transfecting up to 1 ug of mRNA per 200,000 HeLa cells for both MSLN and A*02, surface levels were varied from zero to ~1 million molecules/cell, as determined by cell-surface-Ag quantification (QIFIKIT; see *Methods*). There were slight effects of expression of one Ag on the other and we corrected for these differences in subsequent calculations (**Supplementary Figure 1**).

Having established control over Ag levels on the HeLa target cells, we examined how the dose-response of Jurkat cells transduced with Tmod components varied as a function of quantitative differences in Ag input. The MSLN Tmod cells were co-cultured with HeLa target cells to generate a series of dose-response curves (**Figures 2C, D**). We first considered the response of the Tmod cells to variations in blocker Ag level and focused on 2 key parameters: (1) shift in EC50 of the activator as a measure of activating receptor sensitivity; and (2) shift in Emax as a measure of activating receptor signal magnitude (**Figure 1C**).

Over the roughly million-fold range in A*02 blocker Ag input, the EC50 of the MSLN activator shifted ~100x. The maximum signal (Emax) decreased dramatically with higher A*02 Ag levels, and the background activation level (Emin) also decreased. The span of activation (Emax – Emin) shifted ~20x over the range of blocker Ag examined. This suggests that in MSLN Tmod cells the blocker exerts its effect both by lowering Emax and raising EC50. As the ratio of B-Ag:A-Ag increases, it is more difficult to activate the Jurkat cells fully as a population. For comparison, in human and mouse tissues, MHC-I paralogs average ~50,000 molecules/cell (18, 19). MSLN is expressed at a similar level in normal lung tissue (11). The blue line in **Figure 2C** roughly corresponds to A*02 expression in human tissues, with B-Ag (A*02) expressed on the order of 100,000 copies/cell and MSLN titrated from 0 to ~1,000,000 copies/cell. Compared to the purple line, representative of a MSLN CAR-T that does not distinguish tumor from normal cells, the blue line has a span of activation that is less than half the magnitude (**Figure 2C**), even at the maximum MSLN level of >1,000,000 copies/cell (see **Figure 2A**). This demonstrates that MSLN Tmod functions well at biologically relevant Ag-levels.

This robust behavior was reinforced by examination of the response of the blocker to different amounts of MSLN Ag (**Figure 2D**). Over the roughly 100x range in MSLN levels tested (2-250 ng mRNA; ~3,000-330,000 molecules/cell), the IC50 shifted by ~20x, and the Imin-Imax span changed 7x. At the highest MSLN Ag level studied (~330,000 molecules/cell), the blocker was still able to reduce the maximum activation level by about half at a biologically relevant B-Ag level of ~100,000 molecules/cell (see arrow, **Figure 2D**). Unsurprisingly, a greater number of A*02 Ag molecules were required to block when more MSLN Ag was present; however, this relationship was sublinear. In summary, the MSLN Tmod mechanism displayed a striking degree of control within a wide change of Ag inputs, and the blocker reduced activation by modulating both EC50 and Emax.

#### 3.2.2 KRAS Tmod

**Figure 3:**
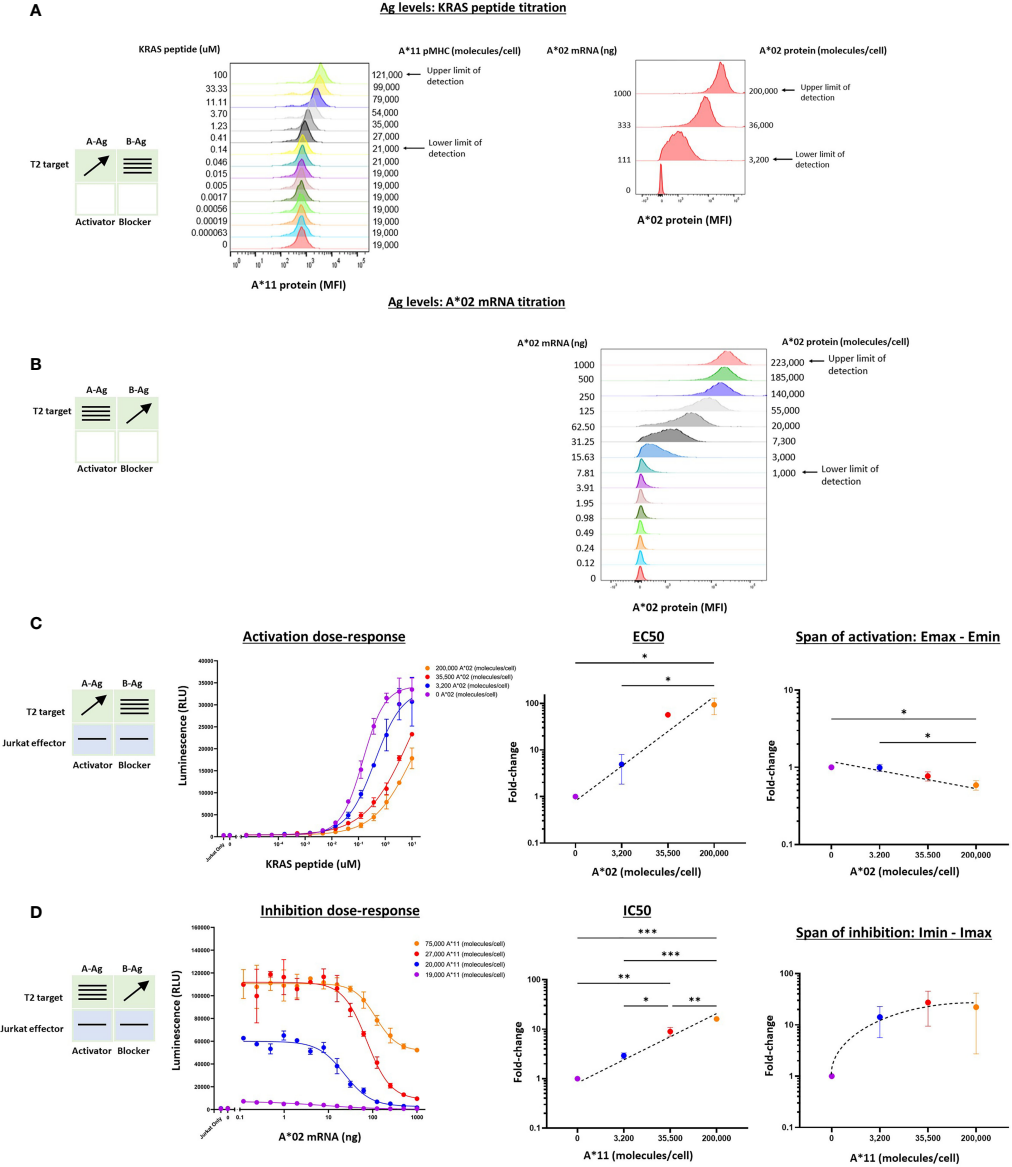
Two-dimensional target Ag/peptide titration (KRAS pMHC and A*02) in T2 [A*11(+)A*02(-)] target cells. 50 μM NY-ESO-1 peptide was added to all cultures to ensure surface expression of A*02 pMHC molecules in T2 cells that are deficient in peptide display (see *Methods*). **(A)** KRAS peptide titrated with A*02 mRNA held constant. A*11 staining is shown as a proxy for KRAS peptide bound to A*11. KRAS peptide titration does not affect A*02 expression. **(B)** A*02 titrated with constant KRAS peptide concentration. A*02 standard curve for mRNA titration in T2 cells are in **Supplementary Figure 2**. The effect of A*02 expression on KRAS/A*11 pMHC level (the equivalent of the left panel in **Figure 2B**) was impossible to determine because an A*11-specific antibody compatible with QIFIKIT was not available. **(C)** Dose-response to KRAS and A*02 target Ag variation with constant activator/blocker ratio on effector cells. EC50 and Emax vs. B-Ag levels. For curves that did not plateau, the maximum value of the fitted curve was used as the Emax value, an underestimate. **(D)** IC50 and Imax vs. A-Ag levels. Note that EC50 and Emax are underestimated for the two highest A*02 levels due to saturation of the assay. A*11 molecules/cells was used as a proxy for KRAS peptide-loaded A*11 molecules and derived from part **(A)**. Dashed lines are meant to convey trends and are not fit mathematically. *p<0.05, **p < 0.01, ***p < 0.001, ****p < 0.0001, n = 2.

We extended this pharmacologic analysis to the KRAS G12V TCR activator with the same A*02 blocker. We used Jurkat effector cells co-cultured with T2 cells genetically modified by HLA-A, -B, and -C knockout and A*11 transgene expression (see *Methods*). Because these modified T2 cells are A*02(-)KRAS G12V(-), we could control Ag levels by titrating the KRAS peptide to exogenously load A*11 molecules, and transfecting the cells with A*02 mRNA. This method is effective because T2 cells are deficient in endogenous peptide display and express only a low level of correctly folded HLA class I molecules on their cell surface (20). To ensure that the A*02 blocker Ag was expressed proportionally to exogenous titrated mRNA, we added 50 μM NY-ESO-1 peptide to specifically bind and stabilize the A*02 molecules on the T2 cell surface (**Supplementary Figure 2B**) (21). The two-dimensional titration experiments were conducted with the same logic used for the MSLN Tmod construct above. In these target cells, the A*02 levels ranged up to ~200,000 molecules/cell (**Figure 3B** and **Supplementary Figure 2C**). The absolute level of KRAS/A*11 was more difficult to estimate, but based on the quantification of A*11 expression and comparisons with other peptide titrations in T2 cells, we estimated a spread from 0 to ~80,000 molecules/cell at maximal peptide-loading of 10 μM (**Figure 3A**, 21).

Co-culturing KRAS Tmod cells with T2 target cells resulted in dose-response curves that enabled estimation of pharmacologic parameters (**Figures 3C, D**). Due to precipitation at higher peptide concentrations, we were not able to interrogate KRAS pMHC levels above 10-100 μM and the dose-response activation curves failed to plateau (**Figure 3C**). Thus, the EC50 and Emax values derived from these curves were likely underestimates, but did not affect our conclusions (see below).

For the KRAS TCR, the EC50 shifted dramatically, ≥100x over the smaller range examined (3,200 – 200,000 A*02 molecules/cell), compared to 100x over a range of 3,000 – 1,000,000 A*02 molecules/cell for the MSLN CAR. Emax was more difficult to assess given the right-shift of the curves into a region where dose-response curves did not plateau. However, from the data points and curves it was clear that Emax – Emin shifts were ≤2x, suggesting that for KRAS Tmod the blocker may control activation more through its effect on activator sensitivity (EC50).

With respect to blocker behavior as a function of KRAS/A*11 input variation, KRAS Tmod was similar to MSLN Tmod. IC50 shifted less than EC50, ~15x across the range of ~75,000 A*11 molecules/cell (10 μM KRAS peptide) tested and the Imin-Imax increased ~22x. At this highest A-Ag level, the blocker exerted detectable inhibition at <20,000 molecules/cell (see arrow, **Figure 3D**). The span of inhibition (Imin – Imax) appeared to plateau at higher levels of A-Ag (**Figure 3D**, far right). This suggests that at very high levels of A-Ag, blocking is not as complete. To summarize, MSLN and KRAS Tmod constructs displayed pharmacologic similarities but with the potential difference that inhibition of the TCR was driven more by EC50 modulation than Emax modulation (see Discussion). This was most obvious in the two-dimensional titrations for activation, as opposed to inhibition, dose-response.

#### 3.2.3 MSLN Tmod in Primary T Cells

**Figure 4:**
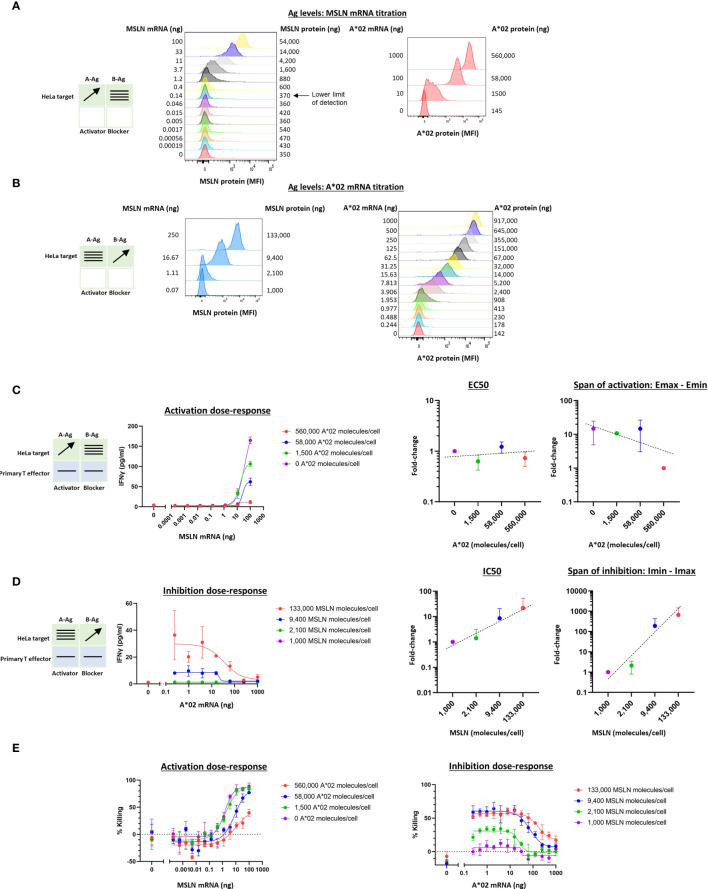
Two-dimensional target Ag (MSLN and A*02) mRNA titration in HeLa [A*02(-)MSLN(-)] target cells with primary T cell effectors. Target cells were a HeLa variant with MSLN knocked out and GFP introduced *via* transduction (see *Methods*). **(A)** MSLN (A-Ag) titration and surface expression visualized by flow cytometry. **(B)** A*02 (B-Ag) titration. **(C)** Dose-response of MSLN and A*02 target Ag variation with constant activator/blocker ratio on primary T effector cells. MSLN A-Ag titrated with varying A*02 B-Ag levels held constant (EC50 and Emax vs B-Ag levels). IFNγ release was measured after 24 hours of co-culture. Fold-change is calculated by normalizing to the lowest value. **(D)** A*02 B-Ag titrated with varying MSLN A-Ag levels held constant (IC50 and Imax vs. A-Ag levels). IFNγ release was measured after 24 hours of co-culture. Fold-change is calculated by normalizing to the lowest A-Ag value. **(E)** Acute killing of GFP-labeled target cells after 12 hours of co-culture. Dashed lines are meant to convey trends and are not fit mathematically.

In previous work we noted a general correlation between activator and blocker receptor function in Jurkat and primary T cells (5, 11, 13, 15). We next attempted to confirm our findings from Jurkat effector cells in primary T cells, focusing on the MSLN CAR/A*02 blocker pair. As before, we used the same HeLa MSLN KO cell line, this time labeled with GFP to enable image-based quantification of target cell killing (see *Methods 2.9*), and titrated either MSLN (**Figure 4A**) or A*02 (**Figure 4B**) while introducing constant amounts of the other Ag. MSLN CAR or MSLN CAR/A*02 blocker were introduced into PBMC-derived primary T cells from two donors using lentiviral transduction (**Supplementary Figure 4**). As in the Jurkat cell experiments, activation or inhibition dose-response curves were generated by co-culturing primary T cells either with (i) target cells transfected with varied amounts of MSLN mRNA and constant amounts of A*02 (**Figure 4C**), or (ii) target cells titrated with different amounts of A*02 and constant amounts of MSLN (**Figure 4D**). IFNγ release was used for curve-fitting and subsequent analysis, as acute killing was subject to more noise (**Figures 4E, F**).

Compared to the corresponding Jurkat cell experiments (**Figure 2C**), in primary T cells, increasing amounts of B-Ag did not cause a significant shift in EC50 (**Figure 4C**). However, the shift in Emax-Emin over the B-Ag range examined was similar to the Jurkat cell results (~20x). Regarding blocker sensitivity as a function of changing amounts of MSLN A-Ag, the IC50 shift was similar to that observed in the parallel Jurkat experiments (~20x; **Figures 2D**, **4D**). The span of blocking (Imin-Imax) was larger in primary T cells (**Figure 4D**) than in Jurkat cells (**Figure 2D**). Acute killing of target cells after 12 hours of co-culture with primary T cells mirrored cytokine release (**Figure 4E**). Overall, despite differences in the assays and effector cells used, MSLN Tmod showed similar trends in behavior in Jurkat and primary T cells. Of note is the consistency in blocker sensitivity between the two types of effectors, demonstrating the usefulness of Jurkat cells as a predictor of primary T cell behavior in this context.

### 3.3 Tmod Receptor Titration

#### 3.3.1 MSLN CAR

Because the Tmod system utilizes two receptors to activate or block effector cell activity, we also wished to understand the effect of varying the ratio of these receptors on functionality. Even when using a single viral vector to introduce the activator and blocker receptors into effector cells, actual surface expression of each receptor may vary stochastically due to receptor turnover or other mechanisms. For example, it could be surmised that a higher activator:blocker ratio would result in less effective blocking. We therefore investigated the effect of activator:blocker ratio on Tmod efficacy.

**Figure 5:**
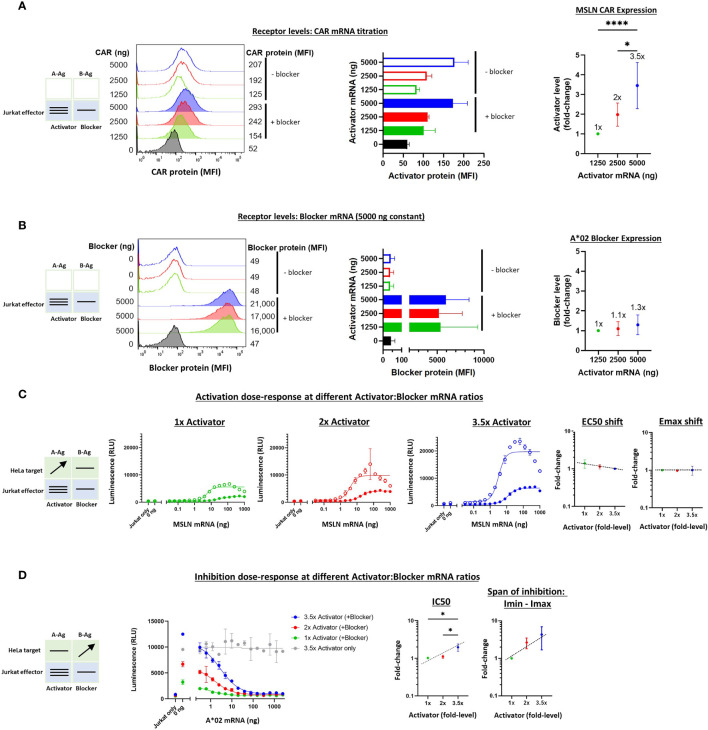
Altering the ratio of MSLN CAR activator to A*02 blocker in effector cells. Cell surface staining of activator and blocker receptors vs. various MSLN CAR mRNA amounts, analyzed by flow cytometry. Blocker was held constant at 5000 ng mRNA and activator (MSLN CAR) was varied at 0, 1250, 2500 and 5000 ng as shown. **(A)** MSLN CAR and A*02 blocker. MSLN CAR was stained with labeled MSLN tetramer (see *Methods*). To calculate fold-expression, background was subtracted before normalization to the lowest value. Bar chart colors match flow histogram colors. **(B)** Blocker protein level is minimally affected by varying activator level. Surface expression of blocker at different mRNA amounts of CAR activator. Blocker was stained with A*02 pMHC tetramer (see *Methods*). Fold-changes shown on graphs (i.e. 1x, 2x, 3.5x) are based on surface protein estimates, not mRNA amount. MFI, geometric mean fluorescence intensity. **(C)** Activation response to varied ratios of MSLN CAR to A*02 blocker. Open circles = CAR only, filled circles = CAR + blocker. Activation dose-response curves are shown for each ratio of CAR to blocker tested. MSLN knockout HeLa cells provided the stimulus. MSLN A-Ag levels were varied with constant B-Ag (2 ng A*02 mRNA, ~3,000 molecules/cell). Shifting of EC50 and Emax with blocker present is shown for each activator amount. **(D)** Inhibition response to varied MSLN CAR to A*02 blocker ratios. Wildtype HeLa cells provided the A-Ag stimulus (endogenously expressed ~50,000 MSLN molecules/cell), and B-Ag (A*02 mRNA) was varied. Dashed lines are meant to convey trends and are not fit mathematically. *p<0.05, **p < 0.01, ***p < 0.001, ****p < 0.0001, n = 2.

To vary the levels of the receptors, as opposed to the Ags, we utilized a similar titration approach as described above. However, receptor mRNAs were transfected into Jurkat cells before co-culturing with the target cells. The signal:noise properties of these assays considerably restricted the range over which different MSLN CAR:A*02 blocker ratios could be tested, and the final ratio range was estimated at 3-4x, based on a combination of measurements that included surface receptor detection *via* MSLN and A*02 tetramer staining as well as Emax values (**Figures 5A, B**, see below). To optimize signal in these experiments, we fixed the blocker mRNA level at 5 ug and varied the amount of MSLN CAR mRNA between 0 and 5 ug per 1.5e6 Jurkat cells. Variation of blocker expression levels was initially tested but subsequently ruled out in this setting because of the production of confounding numbers of activator-only Jurkat cells. We were unable to measure absolute receptor molecules/cell with the detection reagents used, which are incompatible with the QIFIKIT technology, and instead relied on relative values of median fluorescence intensity (see *Methods*).

With the varied receptor expression ratios in Jurkat cells, we could study the dose-response by introducing HeLa target cells as we did above in the Ag-input studies. In these experiments, we performed one-dimensional titration of A-Ag or B-Ag, keeping the other Ag level constant. These experiments generated dose-response curves that enabled assessment of the sensitivity of MSLN Tmod to relative receptor levels. The EC50s were fairly constant, as were the EC50 shifts induced by the blocker, over the ~3.5x difference in activator expression (**Supplementary Figure 5C** and **Figure 5C**). The differences in Emax – Emin were larger, roughly 3-fold, which affirmed a dominant effect on Emax for MSLN Tmod, similar to what was observed in the Ag-titration (**Supplementary Figure 5C**). However, the Emax shifts induced by the blocker were relatively constant amongst all activator:blocker ratios, suggesting that Tmod response is buffered over this span of receptor level ratios (**Figure 5C**).

On the inhibitory side, IC50s and Imin-Imax increased ~2x and ~3x, respectively, as the activator:blocker ratio increased ~3.5x (normalized to the values produced by the lowest amount of activator; see *Calculations* in *Methods*). These results were also consistent with the Ag-titration experiments, lending support to the concept that blocker action in MSLN Tmod cells occurs *via* both Emax and EC50 modulation. Even at the highest activator:blocker ratio, the blocker was able to suppress the activator to a similar baseline level (**Figure 5D**). We did not observe signs of functional instability in the MSLN Tmod system within the range explored.

#### 3.3.2 KRAS TCR

**Figure 6:**
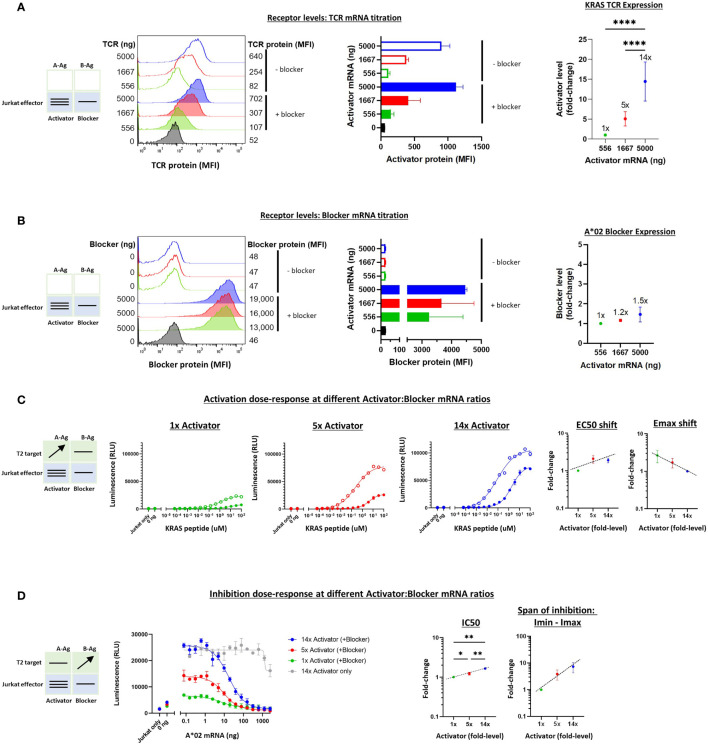
Altering the ratio of KRAS TCR activator to A*02 blocker in effector cells. A*02 blocker mRNA was fixed at 5000 ng and KRAS TCR mRNA was varied at 0, 556, 1667 and 5000 ng as indicated. **(A)** TCR expression was measured by staining with labeled muTCRβ mAb (see *Methods*). **(B)** Surface expression of blocker at different mRNA amounts of CAR activator; blocker protein level was minimally affected by varying activator level. Blocker was stained with A*02 pMHC tetramer (see *Methods*). Fold-changes shown on graphs (i.e. 1x, 2x, 3.5x) are based on surface protein estimates, not mRNA amount. MFI, geometric mean fluorescence intensity. **(C)** Activation response to varied ratios of KRAS TCR to A*02 blocker. Open circles = TCR only, filled circles = TCR + blocker. Activation dose-response curves are shown for each ratio of TCR to blocker tested, titrating A-Ag (KRAS peptide) with constant B-Ag (200 ng A*02 mRNA, ~250,000 molecules/cell). Shifting of EC50 and Emax with blocker present is shown for each activator amount. **(D)** Inhibition response to varied KRAS TCR to A*02 blocker ratios. A-Ag was kept constant (20 nM KRAS peptide) while B-Ag was varied (A*02 mRNA). Dashed lines are meant to convey trends and are not fit mathematically. *p<0.05, **p < 0.01, ***p < 0.001, ****p < 0.0001, n = 2.

To extend these studies to the TCR system, we carried out a similar series of experiments on the KRAS Tmod construct with varied receptor levels. We first tested the quantitative relationship between added synthetic mRNA and receptor expression levels in Jurkat cells. Partly because the background (tonic) signaling of the KRAS TCR was lower than the MSLN CAR, we were able to explore a wider range of activator:blocker ratios, ~14x (**Figures 6A, B**). Because the KRAS TCR contains mouse constant regions in its α/β chains, we used a muTCR mAb to detect its expression on the surface of Jurkat cells. As with MSLN Tmod, we maintained the A*02 blocker at a fixed level to avoid production of activator-only Jurkat cells.

The addition of T2 target cells enabled estimation of the functional effects of receptor ratio differences (**Figures 6C, D**). As with the MSLN CAR, the EC50 and Emax-Emin shifts were relatively small (~2-3x) across different activator:blocker receptor ratios. IC50s and Imin-Imax changed ~2x and ~10x, respectively. Altogether, these results were consistent with the previous experiments regarding shifts in the sensitivity and maximal effect as well as the overall stability of Tmod responses.

#### 3.3.3 MSLN Tmod Receptor Titration in Primary T Cells

**Figure 7:**
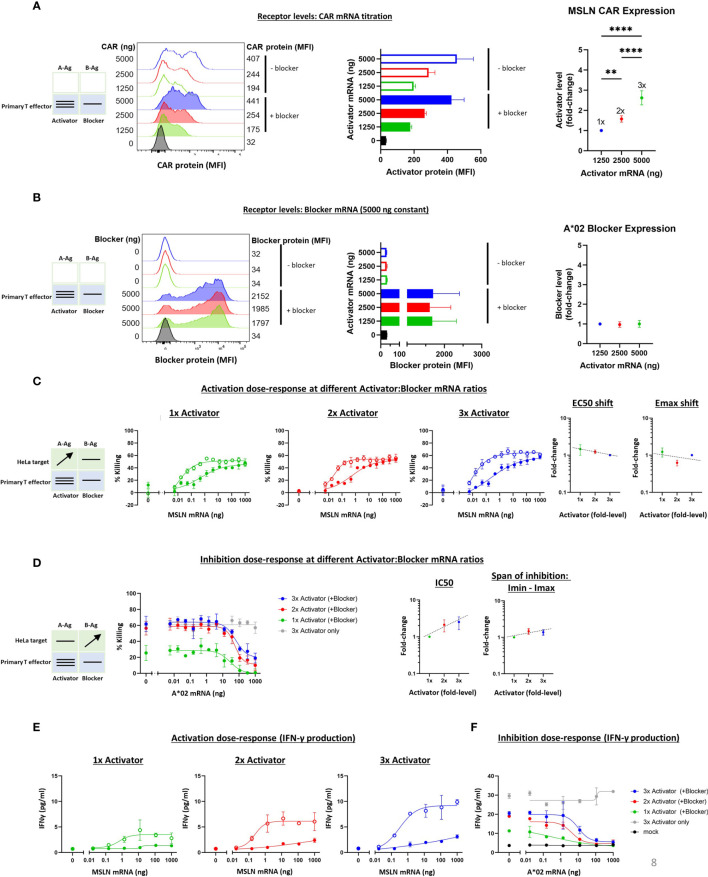
Altering the ratio of MSLN CAR activator to A*02 blocker in primary T effector cells (Donor 1). Cell surface staining of activator and blocker receptors vs. various MSLN CAR mRNA amounts, analyzed by flow cytometry. Blocker was held constant at 5000 ng mRNA and activator (MSLN CAR) was varied at 0, 1250, 2500 and 5000 ng as shown. **(A)** MSLN CAR and A*02 blocker. MSLN CAR was stained with labeled MSLN tetramer (see *Methods*). To calculate fold-expression, background was subtracted before normalization to the lowest value. Bar chart colors match flow histogram colors. **(B)** Blocker protein level is minimally affected by varying activator level. Surface expression of blocker at different mRNA amounts of CAR activator. Blocker was stained with A*02 pMHC tetramer (see *Methods*). Fold-changes shown on graphs (i.e., 1x, 2x, 3x) are based on surface protein estimates, not mRNA amount. MFI, geometric mean fluorescence intensity. **(C)** Activation (killing of GFP-labeled target cells) response to varied ratios of MSLN CAR to A*02 blocker after 12 hours of co-culture with target cells. Activation dose-response curves are shown for each ratio of CAR to blocker tested. Open circles = CAR only, filled circles = CAR + blocker. MSLN knockout HeLa cells (GFP-labeled) provided the stimulus. MSLN A-Ag levels were varied with constant B-Ag (50 ng A*02 mRNA, ~58,000 molecules/cell). Shifting of EC50 and Emax with blocker present is shown for each activator amount. **(D)** Inhibition response to varied MSLN CAR to A*02 blocker ratios after 12 hours of co-culture with target cells. Wildtype HeLa cells (GFP-labeled) provided the A-Ag stimulus (endogenously expressed ~50,000 MSLN molecules/cell), and B-Ag (A*02 mRNA) was varied. Dashed lines are meant to convey trends and are not fit mathematically. **(E, F)** IFNγ release by primary T cells after 24 hours of co-culture for each ratio of CAR to blocker tested. *p<0.05, **p < 0.01, ***p < 0.001, ****p < 0.0001, n = 2.

To extend the receptor titration results from Jurkat cells, we tested MSLN Tmod constructs in primary T cell cytotoxicity and cytokine secretion assays. As in the Jurkat experiments, the A*02 blocker was held constant and MSLN CAR levels were varied by mRNA transfection of primary T cells from 2 donors. A change in activator:blocker mRNA ratio of ~4x resulted in a surface receptor ratio difference of ~3x, with the blocker surface expression verified as roughly constant (**Figures 7A, B** and **Supplementary Figures 9A, B**). A significant percentage of the T cell population did not express the activator and/or the blocker, resulting in a more heterogeneous population of effector cells compared to the corresponding Jurkat cell experiments. We believe these activator-negative cells may affect the signal:noise ratio of the experiment, but not the conclusions.

The various titrations showed a cytotoxicity dose-response from MSLN and A*02 level changes similar to that observed with Jurkat cells (**Figures 7C, D** and **Supplementary Figures 9C, D**). The span of EC50 and Emax shifts was also similar for all receptor ratios tested. In primary T cells from Donor 1, The IC50 varied ~3x over the 3x range, while the Imin-Imax span of change was nearly constant (**Figure 7D**). The Imax for primary T cells expressing 2x and 3x Activator levels was higher than in the parallel Jurkat cell experiments (**Figures 7D**, **5D**), suggesting incomplete blocking. However, A-Ag expression is endogenous and B-Ag expression is transient in these target cells, and this, in addition to bimodal blocker expression in effector cells, is the likely cause of the higher Imax. Furthermore, the primary T cell data shown was collected after 12 hours of co-culture, or ~36 hours after transfection, versus 24 hours after transfection in the Jurkat cell assays; at this later timepoint, the transient B-Ag expression is likely on the decline. Indeed, in previous studies using transduced effector cells and target cells with endogenous A- and B-Ag expression, we observed complete blocking (11). IFNγ secretion in these assays mirrored cytotoxicity (**Figures 7E, F**). Our analysis focuses on the 12-hour timepoint of co-culture, but the cytotoxicity assay displayed consistent trends at different timepoints during the coculture (**Supplementary Figure 8**). As in the Ag titration experiments, these primary T cell results for receptor titration align with the Jurkat cell data for the MSLN CAR and A*02 receptor pair, despite the differences in effector cell and assay types, and support the view that the Tmod system is quite stable with regard to small changes in receptor level ratio.

## 4 Discussion

In this study, we set out to interrogate the robustness and stability of the Tmod system by altering its inputs, namely the Ag and receptor expression levels, and observing the effects on key parameters. This is important because therapeutic Tmod effector cell populations may express variable levels of activator and blocker receptors and will certainly encounter target cells with variable levels of target Ags. Two Tmod constructs were tested, one with MSLN CAR and another with KRAS TCR, each paired with the same A*02 blocker. Importantly, for both Tmod systems tested, the blocker sensitivity (i.e., IC50) only changed ~10-20-fold across a larger range of A-Ag expression levels. Furthermore, despite the extremely large range of Ag levels studied (some up to ~1 million-fold), the resulting changes observed in the parameters (EC50, Emax-Emin, IC50, Imin – Imax) were sublinear. These findings demonstrate the stability of activator and blocker sensitivity when challenged with differing levels of A- and B-Ag, potentially relevant to Tmod function in evolving tumor landscapes and different tissue contexts.

In the analogous receptor titration experiments, we looked for changes in activator and blocker sensitivities upon varying the expression of the receptor(s) on the cell surface. Changing MSLN CAR expression did not affect EC50 but did affect Emax, while changing KRAS TCR expression altered both EC50 and Emax. Similar to our findings from two-dimensional Ag titration in target cells, it was striking that, in both Tmod systems, increasing the activator:blocker receptor ratio did not have a significant effect on the sensitivity of the blocker receptor (i.e., EC50 shift, IC50). Put simply, the blocker functioned similarly well with high and low levels of activator expression. Given the potential complications that could arise from the interplay between two receptors and two Ags, it is remarkable how robustly the blocker functions over a range of Ag and receptor inputs. Altogether, the results from our Ag and receptor titration experiments instill confidence in the potency and stability of the Tmod system.

**Figure 8:**
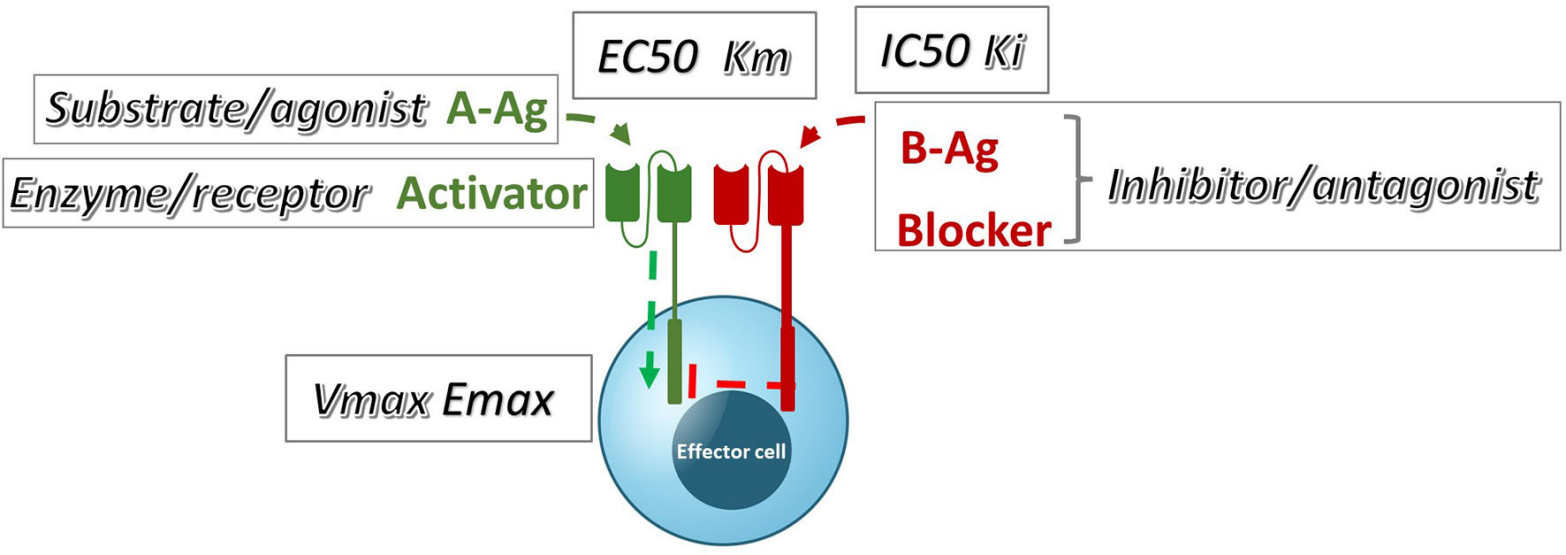
A qualitative comparison of pharmacologic Tmod parameters measured in Jurkat cells and their corresponding analogous Michaelis-Menten or receptor signaling parameters.

These data can also be examined in the context of the conventional pharmacology of antagonism; specifically, they can be compared to well-known models of inhibition (22, Lehninger Principles of Biochemistry, 7^th^ edition). If we group the blocker Ag plus its receptor into a single element of inhibitor/antagonist, there is a plausible and direct analogy with these models among the observed and theoretical parameters (**Figure 8**). By interpreting our data with this framework in mind, we can infer potential pharmacologic mechanisms underlying Tmod blocking.

Perhaps unsurprisingly, the MSLN and KRAS Tmod systems fit best into a mixed model of inhibition/antagonism (**Table 3**). The A*02 blocker controls activation through its effect on both EC50 and Emax. In previous work on activator/blocker pairs that involve pMHCs as target Ags, and TCRs and pMHC-directed CARs as Tmod components, we observed blocking activity with substantial shifts in EC50 (5), consistent with the more detailed analysis performed here. EC50 shifts are diagnostic of competitive inhibitors; thus, the blocker/B-Ag may compete with the A-Ag for a site(s) on the activator that plays a key role in signaling. Emax shifts, on the other hand, are characteristic of other types of antagonism and suggest more complex blocker interactions. This mixed pharmacology is in line with the well-known involvement in TCR signaling of multiple kinases, phosphatases and scaffold proteins, and formation of multi-component structures in the membrane (i.e. the immune synapse; see for review 23, 24). Notably, two different classes of activator, a CAR and a TCR, were studied here. Both displayed similar pharmacology, with the possible exception that the TCR/blocker interaction may be more reminiscent of competitive antagonism (more EC50 shifting than the MSLN/blocker pair). Whether other Tmod activator/blocker pairs fit the same profile remains to be seen. It will also be interesting to explore the range of activator/blocker behaviors using more complex analytical approaches than those employed here (25).

**Table 3:** Observed vs. theoretical behavior (by model type) of Tmod system according to pharmacologic models.

	Experimental change >	↑ B-Ag (inhibitor)	↑ B-Ag (inhibitor)	↑ A-Ag (substrate/agonist)
	**Output >**	**Emax**	**EC50**	**IC50**
**Observed change (Ag titration)**	**MSLN A-Ag**	**↓**	**↑**	**↑**
	**KRAS A-Ag**	**↔/↓**	**↑↑**	**↑**
**Theoretical change**	**Competitive**	**↔**	**↑**	**↑**
	**Uncompetitive (binds ES)**	**↓**	**↓**	**↓**
	**Non-competitive (allosteric)**	**↓**	**↔**	**↔**

The experimental changes are: increased B-Ag (analogous to increased inhibitor) or increased A-Ag (analogous to increased substrate/agonist).

These findings are subject to various caveats. The data reported here are limited to a few receptor/Ag pairs; we expect and have begun to confirm that others work similarly. For technical reasons we were able to explore only a restricted range of receptor levels compared to Ag levels. Although we have previously noted a strong correlation between many quantitative measurements in Jurkat and primary T cells, the experiments here were focused around Jurkat cells with more limited study of primary T cells, the relevant therapeutic entity. Also, we measured population-averaged behavior and, were we to study the effects on single cells, some important mechanistic detail might emerge; for example, analog vs. digital behavior of the system. Finally, we did not examine the biochemistry and biophysics that may underlie some of the mechanisms discussed. These remain fruitful areas of future investigation.

The field is in the early stages of understanding the mechanisms underlying artificial cell-surface signal integrators such as the Tmod system. Given the complexity and extraordinary properties of TCR signaling itself, it is not surprising that a great deal of mechanistic detail remains to be unraveled regarding Tmod function (26). The Tmod constructs studied here employ a LIR-1-based blocker redirected specifically to A*02. LIR-1 is believed to function through ligand-dependent interaction of its ITIM domains with activating receptors, in this case the TCR and CAR (27). This interaction shifts the balance of protein phosphorylation to disfavor the activation signal. It is also known that CARs are subject to down-modulation of surface levels (28–30). We have evidence that the Tmod blocker may function in part by regulating the surface levels of the activator and these results will be presented elsewhere. The normal ligands of LIR-1 are related to the A*02 blocker Ag we have studied here, but more numerous: all/most class I paralogous and allelic products (31). Moreover, LIR-1 is expressed either at low levels or in a small subpopulation of T cells, so it seems unlikely that Tmod mimics normal LIR-1-specific biology perfectly (10; The GTEx Project). It is possible that the mechanistic findings discussed here may either be general properties of ITAM/ITIM interactions or unique to the specific LIR-1-scFv blocker in the context of T cells. We do not know, for example, which properties other immune-inhibitory receptors may share with the A*02 blocker. Nonetheless, we believe these pharmacologic data around buffering capacity demonstrate Tmod’s utility as a cancer therapeutic and encourage further investigation.

## Data Availability Statement

The original contributions presented in the study are included in the article/**Supplementary Material**. Further inquiries can be directed to the corresponding authors.

## Author Contributions

DM, BD, and AK contributed to the conception and design of the study. DM, KB, and BD conducted the experiments. DM and KB performed the data analysis. BD and J-YM gave experimental guidance and contributed to study design throughout the process. DM, KB, and AK wrote the manuscript. J-YM assisted with manuscript structuring and provided feedback. All authors contributed to the article and approved the submitted version.

## Conflict of Interest

All authors are employees and shareholders of A2 Biotherapeutics.

## Publisher’s Note

All claims expressed in this article are solely those of the authors and do not necessarily represent those of their affiliated organizations, or those of the publisher, the editors and the reviewers. Any product that may be evaluated in this article, or claim that may be made by its manufacturer, is not guaranteed or endorsed by the publisher.
