# Supplementary material for: Robust In Vitro Pharmacology of Tmod, a Synthetic Dual-Signal Integrator for Cancer Cell Therapy
Source: Front Immunol. 2022 Mar 10;13:826747. doi: 10.3389/fimmu.2022.826747 (PMC8960424; doi:10.3389/fimmu.2022.826747)
Supplement: Supplementary file 1 [file Presentation_1.pptx]

## Slide 1
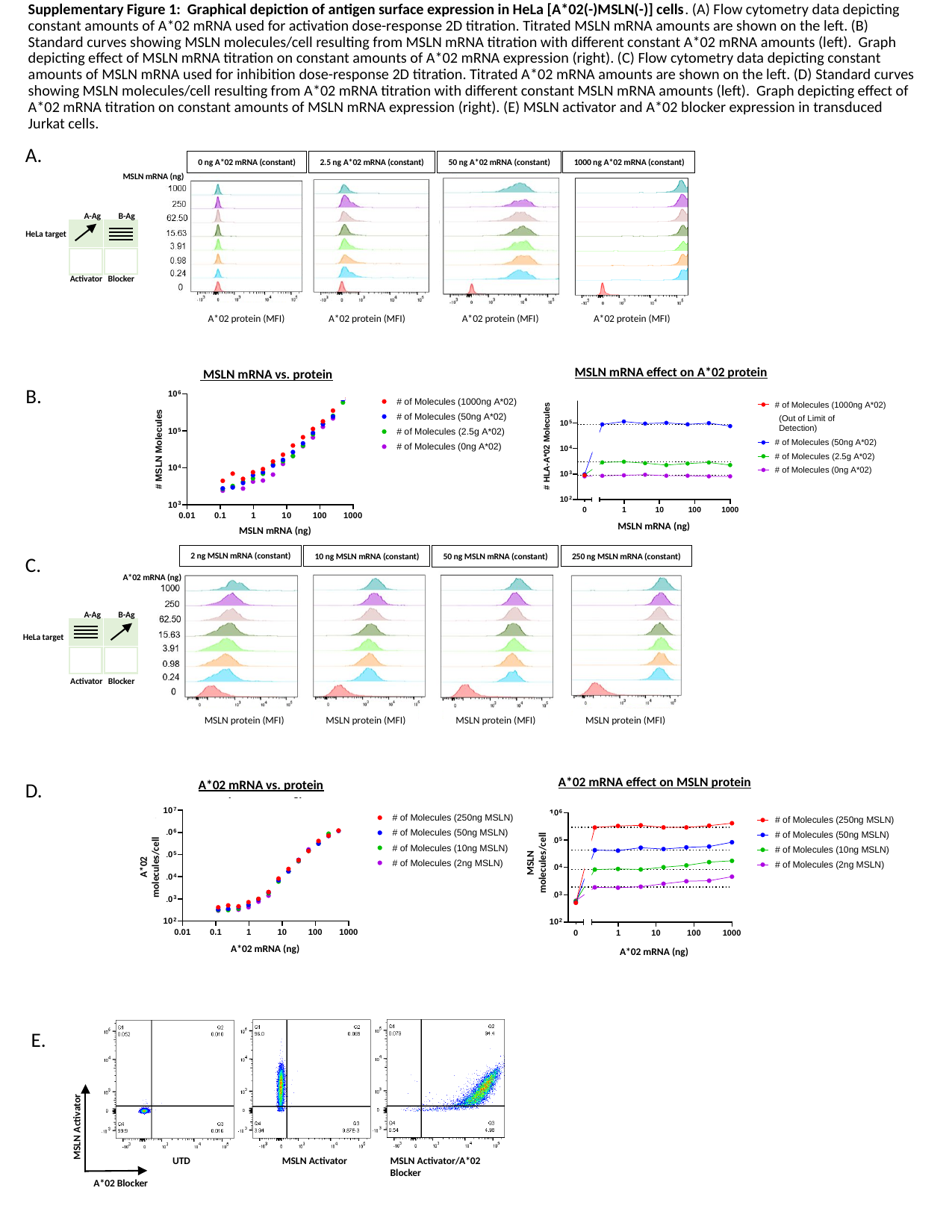

# Supplementary Figure 1: Graphical depiction of antigen surface expression in HeLa [A*02(-)MSLN(-)] cells. (A) Flow cytometry data depicting constant amounts of A*02 mRNA used for activation dose-response 2D titration. Titrated MSLN mRNA amounts are shown on the left. (B) Standard curves showing MSLN molecules/cell resulting from MSLN mRNA titration with different constant A*02 mRNA amounts (left). Graph depicting effect of MSLN mRNA titration on constant amounts of A*02 mRNA expression (right). (C) Flow cytometry data depicting constant amounts of MSLN mRNA used for inhibition dose-response 2D titration. Titrated A*02 mRNA amounts are shown on the left. (D) Standard curves showing MSLN molecules/cell resulting from A*02 mRNA titration with different constant MSLN mRNA amounts (left). Graph depicting effect of A*02 mRNA titration on constant amounts of MSLN mRNA expression (right). (E) MSLN activator and A*02 blocker expression in transduced Jurkat cells.
A.
0 ng A*02 mRNA (constant)
2.5 ng A*02 mRNA (constant)
50 ng A*02 mRNA (constant)
1000 ng A*02 mRNA (constant)
MSLN mRNA (ng)
A*02 protein (MFI)
A*02 protein (MFI)
A*02 protein (MFI)
A*02 protein (MFI)
A-Ag
B-Ag
HeLa target
Activator
Blocker
Molecules vs. mRNA
 MSLN mRNA vs. protein
MSLN mRNA (ng)
MSLN mRNA effect on A*02 protein
MSLN mRNA (ng)
B.
2 ng MSLN mRNA (constant)
10 ng MSLN mRNA (constant)
250 ng MSLN mRNA (constant)
50 ng MSLN mRNA (constant)
A*02 mRNA (ng)
MSLN protein (MFI)
MSLN protein (MFI)
MSLN protein (MFI)
MSLN protein (MFI)
C.
A-Ag
B-Ag
HeLa target
Activator
Blocker
A*02 mRNA effect on MSLN protein
A*02 mRNA vs. protein
D.
MSLN molecules/cell
A*02 molecules/cell
A*02 mRNA (ng)
A*02 mRNA (ng)
E.
MSLN Activator
A*02 Blocker
UTD
MSLN Activator
MSLN Activator/A*02 Blocker

## Slide 2
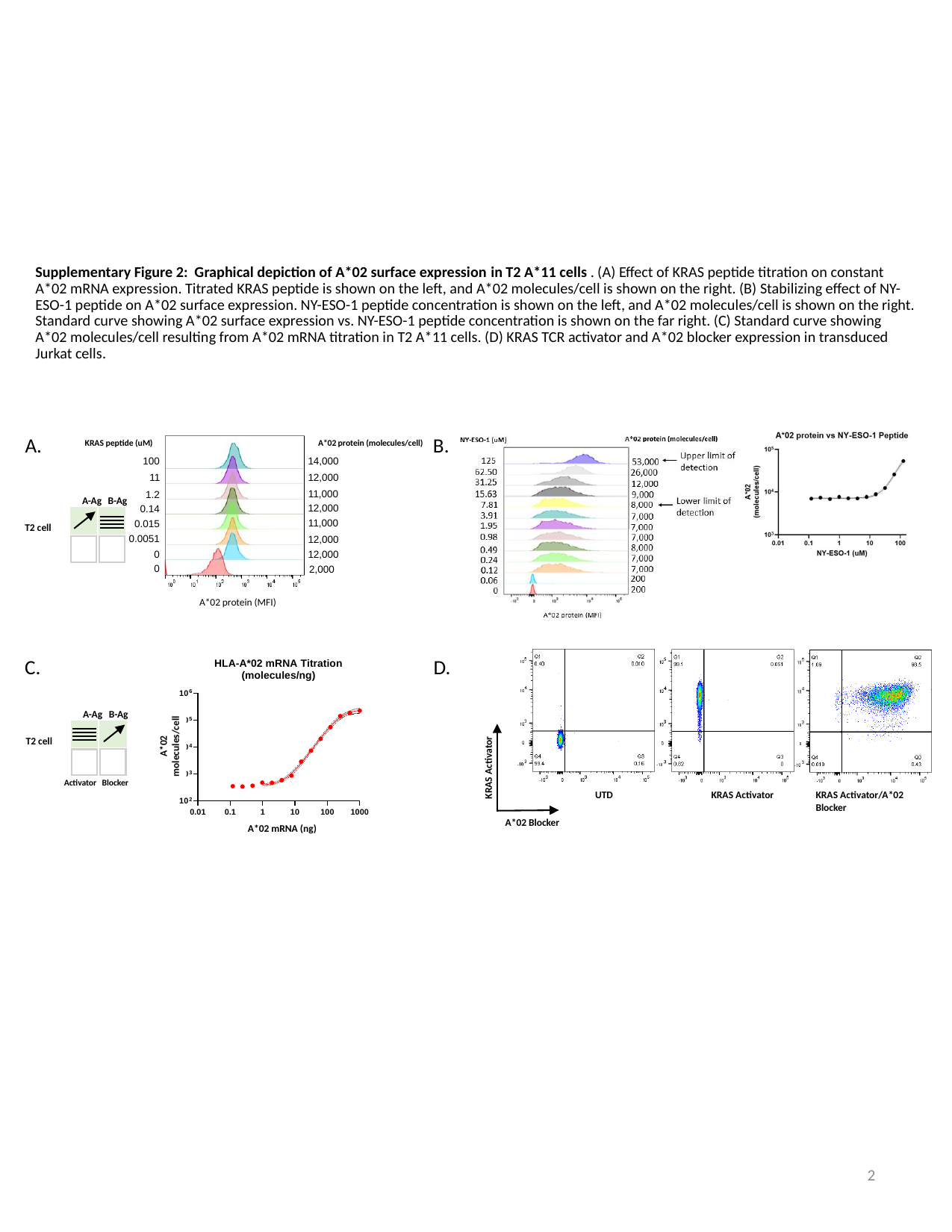

Supplementary Figure 2: Graphical depiction of A*02 surface expression in T2 A*11 cells . (A) Effect of KRAS peptide titration on constant A*02 mRNA expression. Titrated KRAS peptide is shown on the left, and A*02 molecules/cell is shown on the right. (B) Stabilizing effect of NY-ESO-1 peptide on A*02 surface expression. NY-ESO-1 peptide concentration is shown on the left, and A*02 molecules/cell is shown on the right. Standard curve showing A*02 surface expression vs. NY-ESO-1 peptide concentration is shown on the far right. (C) Standard curve showing A*02 molecules/cell resulting from A*02 mRNA titration in T2 A*11 cells. (D) KRAS TCR activator and A*02 blocker expression in transduced Jurkat cells.
A.
B.
KRAS peptide (uM)
A*02 protein (molecules/cell)
14,000
100
11
12,000
11,000
1.2
12,000
0.14
11,000
0.015
0.0051
12,000
0
12,000
0
2,000
A*02 protein (MFI)
A-Ag
B-Ag
T2 cell
C.
D.
A*02 molecules/cell
A*02 mRNA (ng)
KRAS Activator
A*02 Blocker
A-Ag
B-Ag
T2 cell
Activator
Blocker
UTD
KRAS Activator
KRAS Activator/A*02 Blocker
2

## Slide 3
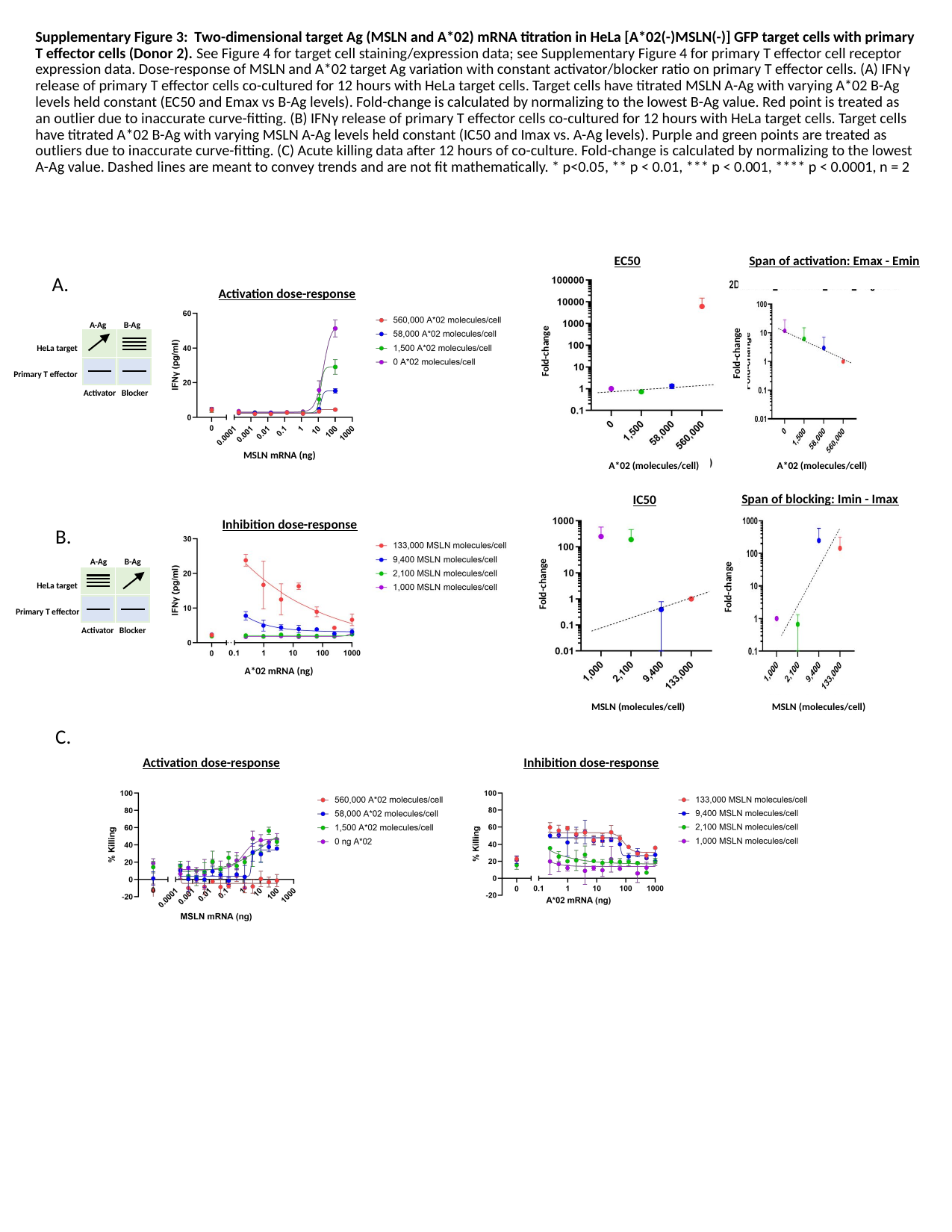

Supplementary Figure 3: Two-dimensional target Ag (MSLN and A*02) mRNA titration in HeLa [A*02(-)MSLN(-)] GFP target cells with primary T effector cells (Donor 2). See Figure 4 for target cell staining/expression data; see Supplementary Figure 4 for primary T effector cell receptor expression data. Dose-response of MSLN and A*02 target Ag variation with constant activator/blocker ratio on primary T effector cells. (A) IFNγ release of primary T effector cells co-cultured for 12 hours with HeLa target cells. Target cells have titrated MSLN A-Ag with varying A*02 B-Ag levels held constant (EC50 and Emax vs B-Ag levels). Fold-change is calculated by normalizing to the lowest B-Ag value. Red point is treated as an outlier due to inaccurate curve-fitting. (B) IFNγ release of primary T effector cells co-cultured for 12 hours with HeLa target cells. Target cells have titrated A*02 B-Ag with varying MSLN A-Ag levels held constant (IC50 and Imax vs. A-Ag levels). Purple and green points are treated as outliers due to inaccurate curve-fitting. (C) Acute killing data after 12 hours of co-culture. Fold-change is calculated by normalizing to the lowest A-Ag value. Dashed lines are meant to convey trends and are not fit mathematically. * p<0.05, ** p < 0.01, *** p < 0.001, **** p < 0.0001, n = 2
EC50
Span of activation: Emax - Emin
A.
Activation dose-response
A-Ag
B-Ag
HeLa target
 Fold-change
 Fold-change
Primary T effector
Activator
Blocker
MSLN mRNA (ng)
 A*02 (molecules/cell)
A*02 (molecules/cell)
Span of blocking: Imin - Imax
IC50
Inhibition dose-response
B.
A-Ag
B-Ag
 Fold-change
HeLa target
 Fold-change
Primary T effector
Activator
Blocker
A*02 mRNA (ng)
MSLN (molecules/cell)
 MSLN (molecules/cell)
C.
Inhibition dose-response
Activation dose-response

## Slide 4
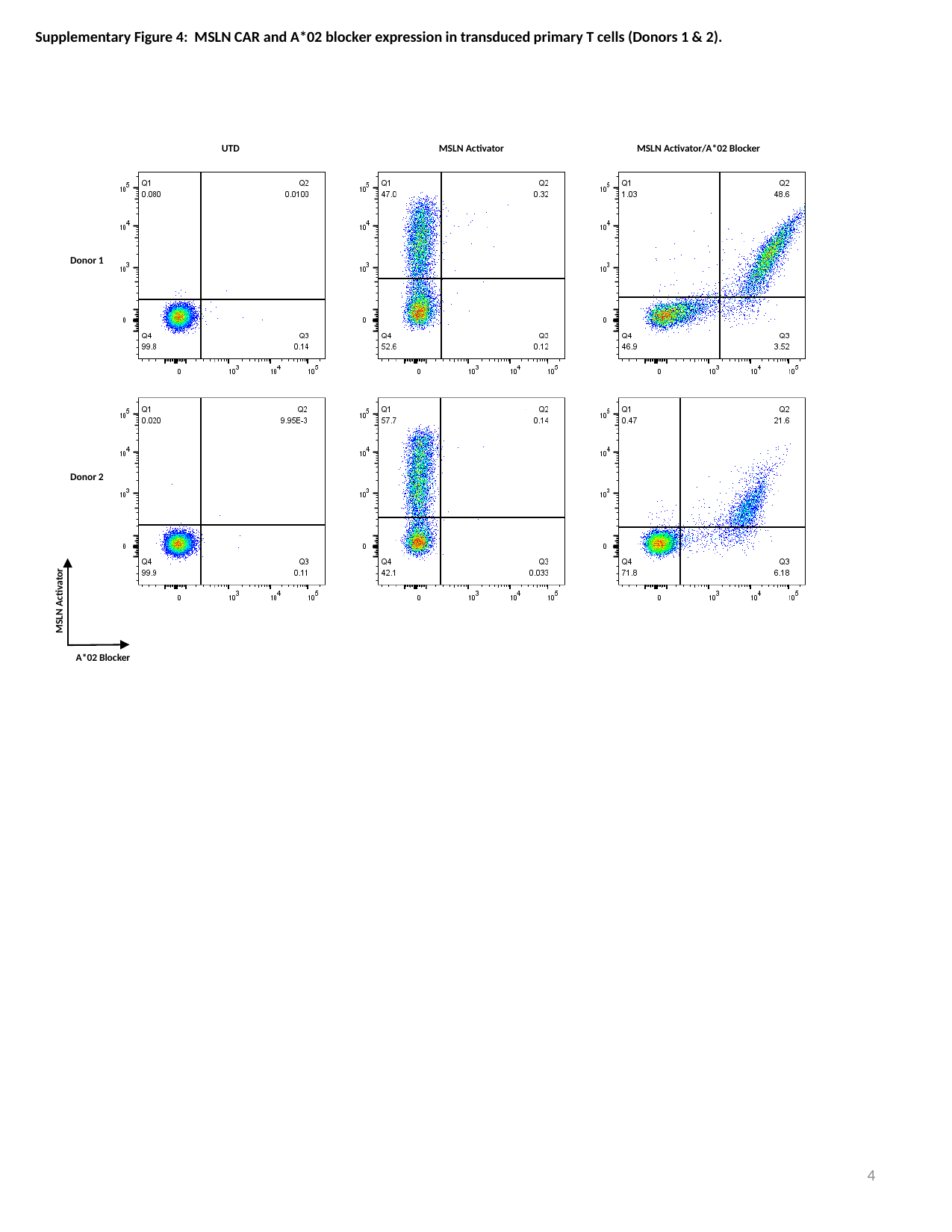

Supplementary Figure 4: MSLN CAR and A*02 blocker expression in transduced primary T cells (Donors 1 & 2).
UTD
MSLN Activator
MSLN Activator/A*02 Blocker
Donor 1
Donor 2
MSLN Activator
A*02 Blocker
4

## Slide 5
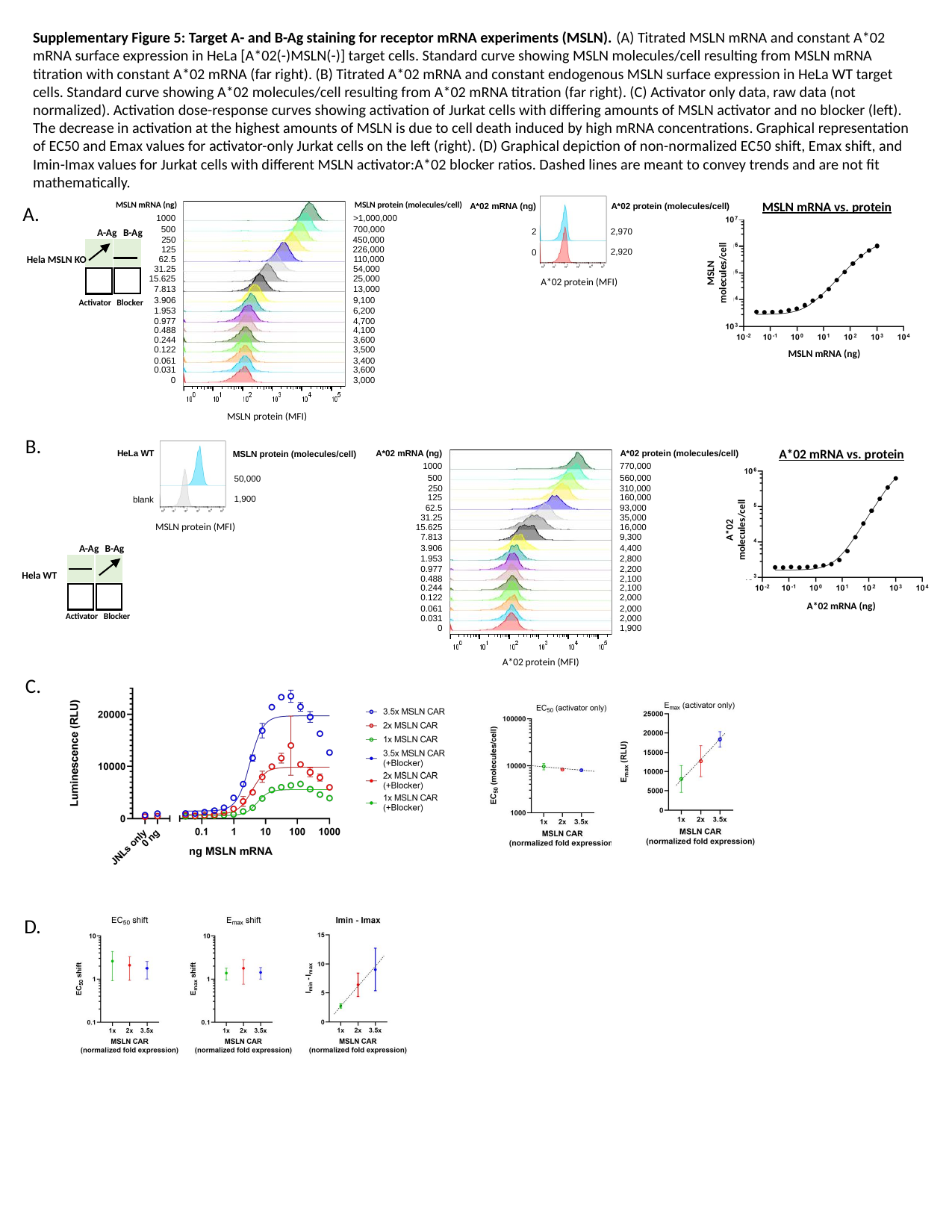

Supplementary Figure 5: Target A- and B-Ag staining for receptor mRNA experiments (MSLN). (A) Titrated MSLN mRNA and constant A*02 mRNA surface expression in HeLa [A*02(-)MSLN(-)] target cells. Standard curve showing MSLN molecules/cell resulting from MSLN mRNA titration with constant A*02 mRNA (far right). (B) Titrated A*02 mRNA and constant endogenous MSLN surface expression in HeLa WT target cells. Standard curve showing A*02 molecules/cell resulting from A*02 mRNA titration (far right). (C) Activator only data, raw data (not normalized). Activation dose-response curves showing activation of Jurkat cells with differing amounts of MSLN activator and no blocker (left). The decrease in activation at the highest amounts of MSLN is due to cell death induced by high mRNA concentrations. Graphical representation of EC50 and Emax values for activator-only Jurkat cells on the left (right). (D) Graphical depiction of non-normalized EC50 shift, Emax shift, and Imin-Imax values for Jurkat cells with different MSLN activator:A*02 blocker ratios. Dashed lines are meant to convey trends and are not fit mathematically.
MSLN mRNA (ng)
MSLN protein (molecules/cell)
1000
>1,000,000
500
700,000
250
450,000
125
226,000
62.5
110,000
31.25
54,000
15.625
25,000
7.813
13,000
3.906
9,100
1.953
6,200
0.977
4,700
0.488
4,100
0.244
3,600
0.122
3,500
0.061
3,400
0.031
3,600
0
3,000
MSLN mRNA vs. protein
MSLN mRNA (ng)
MSLN molecules/cell
A*02 mRNA (ng)
A*02 protein (molecules/cell)
2,970
2
2,920
0
A*02 protein (MFI)
A.
A-Ag
B-Ag
Hela MSLN KO
Activator
Blocker
MSLN protein (MFI)
A*02 mRNA (ng)
A*02 protein (molecules/cell)
1000
770,000
500
560,000
250
310,000
125
160,000
62.5
93,000
31.25
35,000
15.625
16,000
7.813
9,300
3.906
4,400
1.953
2,800
0.977
2,200
0.488
2,100
0.244
2,100
0.122
2,000
0.061
2,000
0.031
2,000
0
1,900
A*02 protein (MFI)
B.
HeLa WT
MSLN protein (molecules/cell)
50,000
1,900
blank
MSLN protein (MFI)
A*02 mRNA vs. protein
A*02 molecules/cell
A*02 mRNA (ng)
A-Ag
B-Ag
Hela WT
Activator
Blocker
C.
D.

## Slide 6
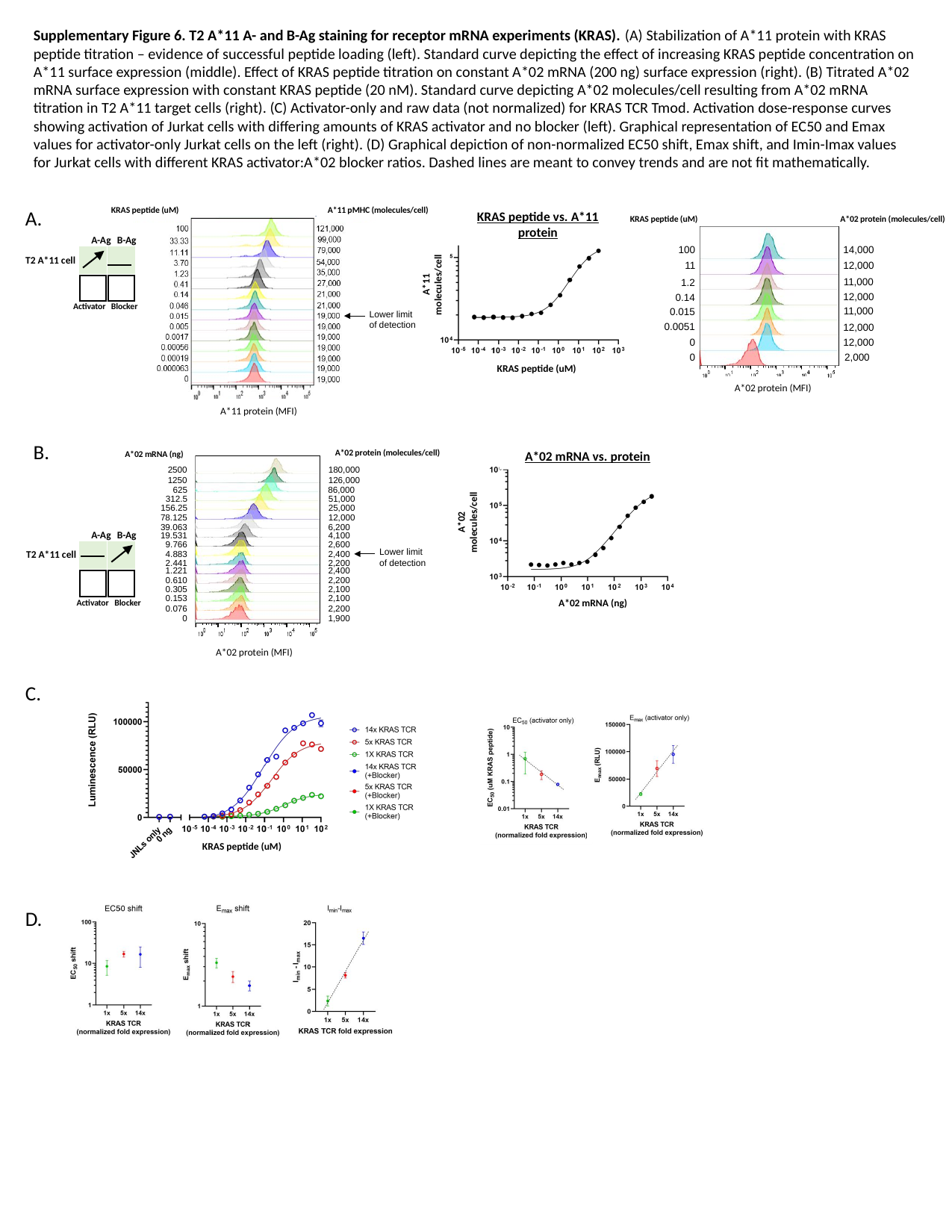

Supplementary Figure 6. T2 A*11 A- and B-Ag staining for receptor mRNA experiments (KRAS). (A) Stabilization of A*11 protein with KRAS peptide titration – evidence of successful peptide loading (left). Standard curve depicting the effect of increasing KRAS peptide concentration on A*11 surface expression (middle). Effect of KRAS peptide titration on constant A*02 mRNA (200 ng) surface expression (right). (B) Titrated A*02 mRNA surface expression with constant KRAS peptide (20 nM). Standard curve depicting A*02 molecules/cell resulting from A*02 mRNA titration in T2 A*11 target cells (right). (C) Activator-only and raw data (not normalized) for KRAS TCR Tmod. Activation dose-response curves showing activation of Jurkat cells with differing amounts of KRAS activator and no blocker (left). Graphical representation of EC50 and Emax values for activator-only Jurkat cells on the left (right). (D) Graphical depiction of non-normalized EC50 shift, Emax shift, and Imin-Imax values for Jurkat cells with different KRAS activator:A*02 blocker ratios. Dashed lines are meant to convey trends and are not fit mathematically.
KRAS peptide (uM)
A*11 pMHC (molecules/cell)
A*11 protein (MFI)
A.
KRAS peptide vs. A*11 protein
A*11 molecules/cell
KRAS peptide (uM)
A*02 protein (molecules/cell)
KRAS peptide (uM)
A-Ag
B-Ag
14,000
100
T2 A*11 cell
11
12,000
11,000
1.2
12,000
0.14
Activator
Blocker
11,000
0.015
Lower limit
of detection
0.0051
12,000
0
12,000
0
2,000
A*02 protein (MFI)
B.
A*02 mRNA vs. protein
A*02 molecules/cell
A*02 mRNA (ng)
A*02 protein (molecules/cell)
A*02 mRNA (ng)
2500
180,000
1250
126,000
625
86,000
312.5
51,000
156.25
25,000
78.125
12,000
39.063
6,200
A-Ag
B-Ag
19.531
4,100
9.766
2,600
Lower limit
of detection
T2 A*11 cell
4.883
2,400
2.441
2,200
1.221
2,400
0.610
2,200
0.305
2,100
0.153
2,100
Activator
Blocker
0.076
2,200
0
1,900
A*02 protein (MFI)
C.
KRAS peptide (uM)
D.

## Slide 7
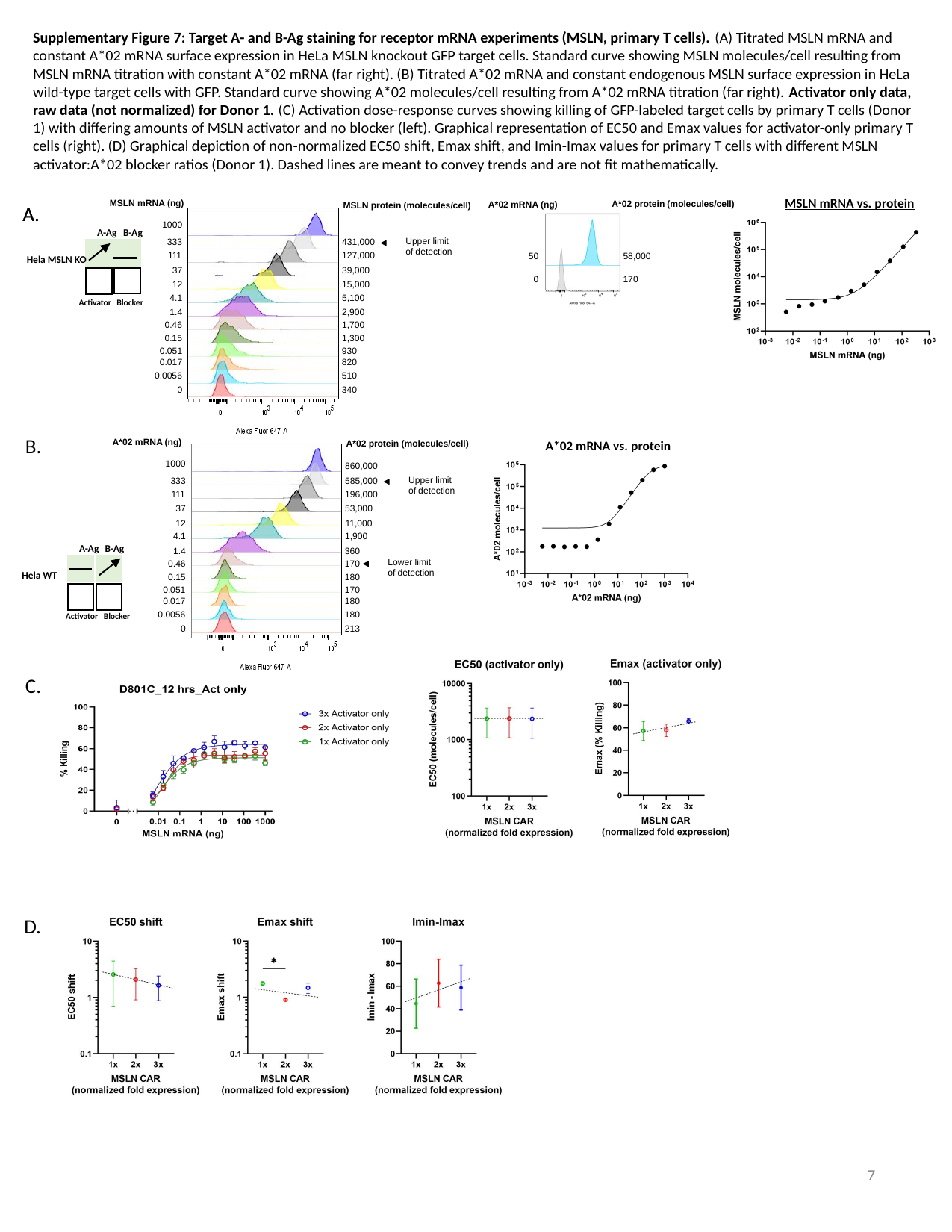

Supplementary Figure 7: Target A- and B-Ag staining for receptor mRNA experiments (MSLN, primary T cells). (A) Titrated MSLN mRNA and constant A*02 mRNA surface expression in HeLa MSLN knockout GFP target cells. Standard curve showing MSLN molecules/cell resulting from MSLN mRNA titration with constant A*02 mRNA (far right). (B) Titrated A*02 mRNA and constant endogenous MSLN surface expression in HeLa wild-type target cells with GFP. Standard curve showing A*02 molecules/cell resulting from A*02 mRNA titration (far right). Activator only data, raw data (not normalized) for Donor 1. (C) Activation dose-response curves showing killing of GFP-labeled target cells by primary T cells (Donor 1) with differing amounts of MSLN activator and no blocker (left). Graphical representation of EC50 and Emax values for activator-only primary T cells (right). (D) Graphical depiction of non-normalized EC50 shift, Emax shift, and Imin-Imax values for primary T cells with different MSLN activator:A*02 blocker ratios (Donor 1). Dashed lines are meant to convey trends and are not fit mathematically.
MSLN mRNA vs. protein
MSLN mRNA (ng)
MSLN protein (molecules/cell)
1000
333
111
37
12
4.1
1.4
0.46
0.15
0.051
0.017
0.0056
0
Upper limit
of detection
431,000
127,000
39,000
15,000
5,100
2,900
1,700
1,300
930
820
510
340
A*02 protein (molecules/cell)
A*02 mRNA (ng)
A.
A.
A-Ag
B-Ag
50
58,000
Hela MSLN KO
0
170
Activator
Blocker
B.
A*02 mRNA (ng)
A*02 protein (molecules/cell)
1000
333
111
37
12
4.1
1.4
0.46
0.15
0.051
0.017
0.0056
0
Upper limit
of detection
585,000
196,000
53,000
11,000
1,900
360
170
180
170
180
180
213
A*02 mRNA vs. protein
860,000
A-Ag
B-Ag
Hela WT
Activator
Blocker
Lower limit
of detection
C.
D.
7

## Slide 8
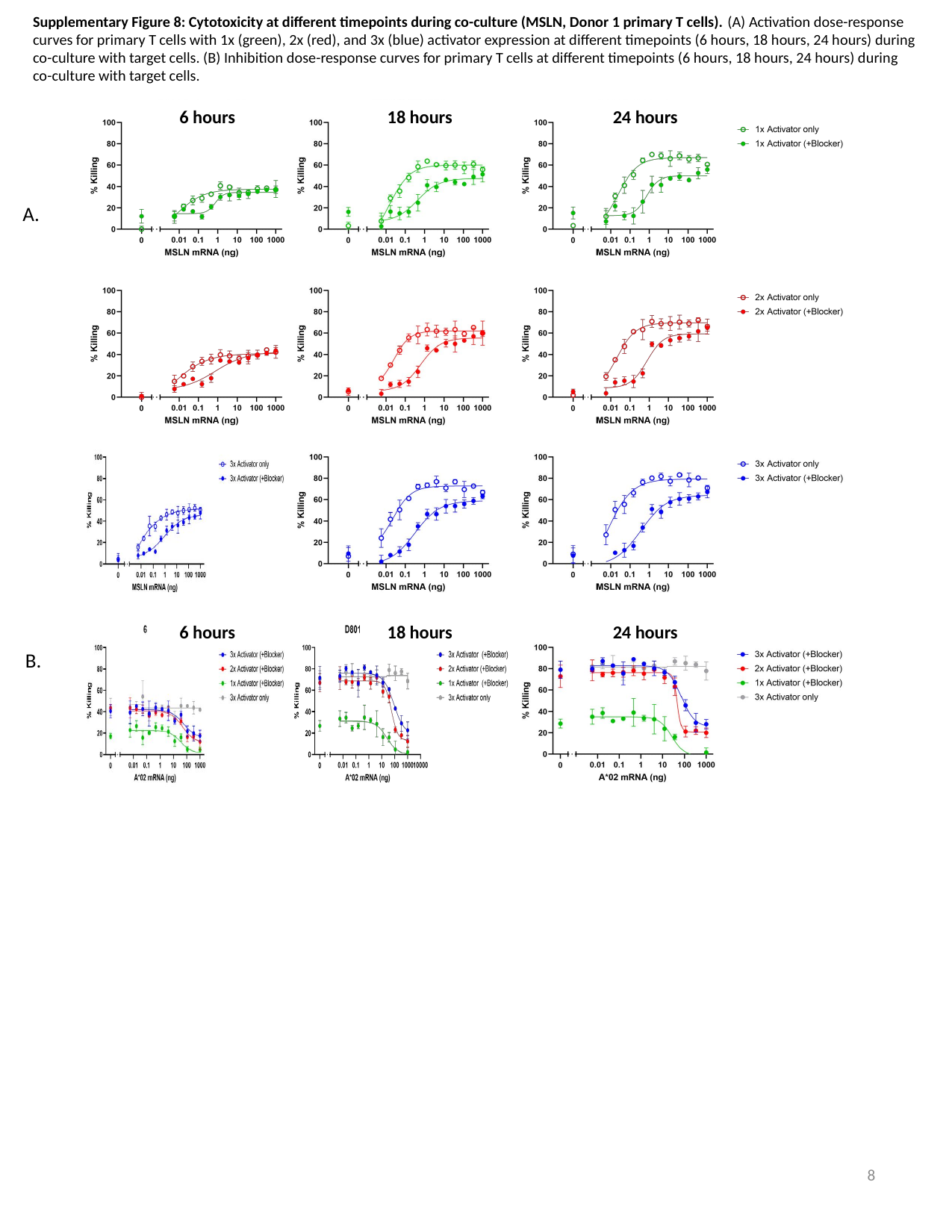

Supplementary Figure 8: Cytotoxicity at different timepoints during co-culture (MSLN, Donor 1 primary T cells). (A) Activation dose-response curves for primary T cells with 1x (green), 2x (red), and 3x (blue) activator expression at different timepoints (6 hours, 18 hours, 24 hours) during co-culture with target cells. (B) Inhibition dose-response curves for primary T cells at different timepoints (6 hours, 18 hours, 24 hours) during co-culture with target cells.
6 hours
18 hours
24 hours
A.
6 hours
18 hours
24 hours
B.
8

## Slide 9
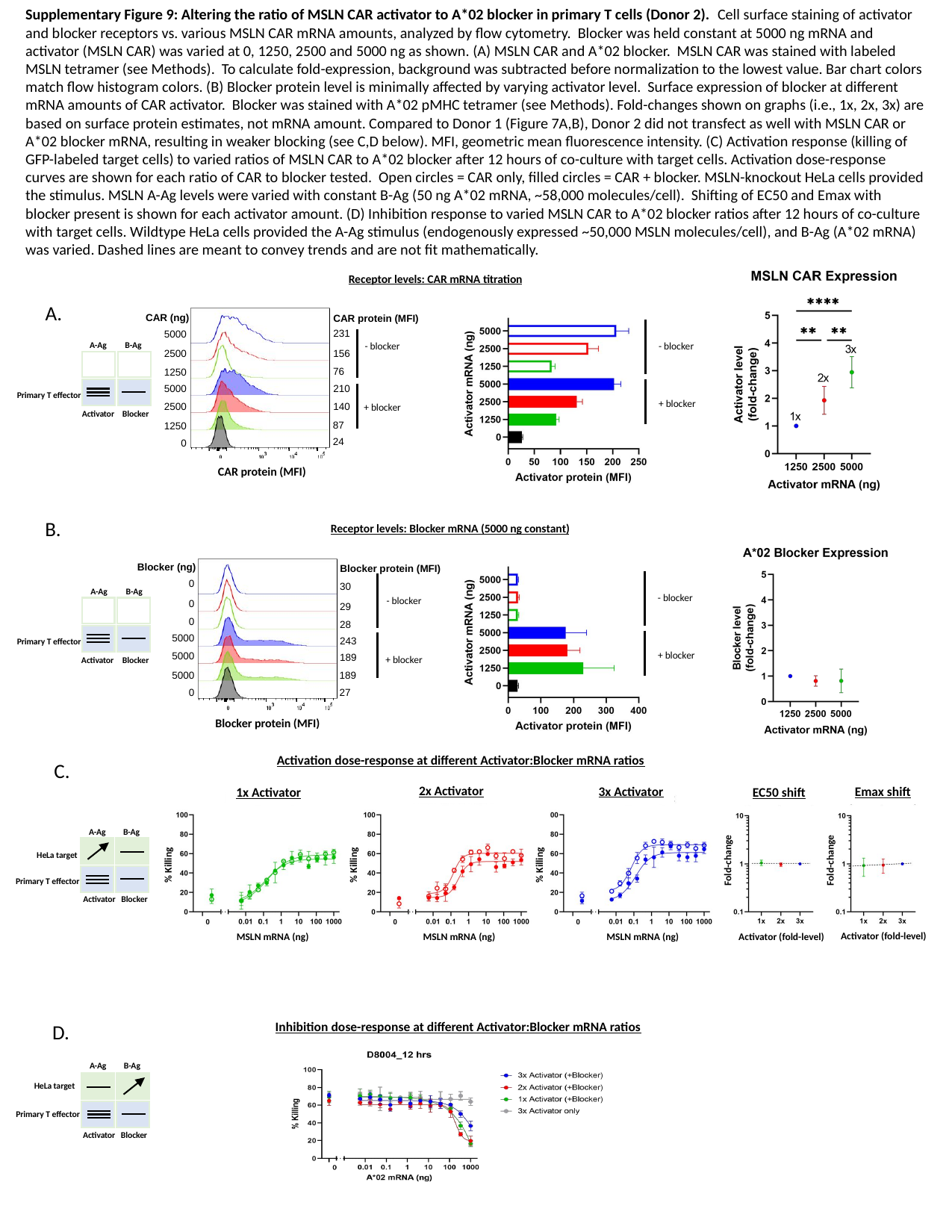

Supplementary Figure 9: Altering the ratio of MSLN CAR activator to A*02 blocker in primary T cells (Donor 2). Cell surface staining of activator and blocker receptors vs. various MSLN CAR mRNA amounts, analyzed by flow cytometry. Blocker was held constant at 5000 ng mRNA and activator (MSLN CAR) was varied at 0, 1250, 2500 and 5000 ng as shown. (A) MSLN CAR and A*02 blocker. MSLN CAR was stained with labeled MSLN tetramer (see Methods). To calculate fold-expression, background was subtracted before normalization to the lowest value. Bar chart colors match flow histogram colors. (B) Blocker protein level is minimally affected by varying activator level. Surface expression of blocker at different mRNA amounts of CAR activator. Blocker was stained with A*02 pMHC tetramer (see Methods). Fold-changes shown on graphs (i.e., 1x, 2x, 3x) are based on surface protein estimates, not mRNA amount. Compared to Donor 1 (Figure 7A,B), Donor 2 did not transfect as well with MSLN CAR or A*02 blocker mRNA, resulting in weaker blocking (see C,D below). MFI, geometric mean fluorescence intensity. (C) Activation response (killing of GFP-labeled target cells) to varied ratios of MSLN CAR to A*02 blocker after 12 hours of co-culture with target cells. Activation dose-response curves are shown for each ratio of CAR to blocker tested. Open circles = CAR only, filled circles = CAR + blocker. MSLN-knockout HeLa cells provided the stimulus. MSLN A-Ag levels were varied with constant B-Ag (50 ng A*02 mRNA, ~58,000 molecules/cell). Shifting of EC50 and Emax with blocker present is shown for each activator amount. (D) Inhibition response to varied MSLN CAR to A*02 blocker ratios after 12 hours of co-culture with target cells. Wildtype HeLa cells provided the A-Ag stimulus (endogenously expressed ~50,000 MSLN molecules/cell), and B-Ag (A*02 mRNA) was varied. Dashed lines are meant to convey trends and are not fit mathematically.
Receptor levels: CAR mRNA titration
A.
CAR (ng)
5000
2500
1250
5000
2500
1250
0
CAR protein (MFI)
231
- blocker
156
76
210
140
+ blocker
87
24
- blocker
+ blocker
A-Ag
B-Ag
Primary T effector
Activator
Blocker
CAR protein (MFI)
B.
Receptor levels: Blocker mRNA (5000 ng constant)
Blocker (ng)
0
0
0
5000
5000
5000
0
Blocker protein (MFI)
30
- blocker
29
28
243
189
+ blocker
189
27
- blocker
+ blocker
A-Ag
B-Ag
Primary T effector
Activator
Blocker
Blocker protein (MFI)
Activation dose-response at different Activator:Blocker mRNA ratios
C.
2x Activator
3x Activator
Emax shift
EC50 shift
1x Activator
A-Ag
B-Ag
HeLa target
Primary T effector
Activator
Blocker
Fold-change
Fold-change
% Killing
% Killing
% Killing
Activator (fold-level)
Activator (fold-level)
MSLN mRNA (ng)
MSLN mRNA (ng)
MSLN mRNA (ng)
Inhibition dose-response at different Activator:Blocker mRNA ratios
D.
A-Ag
B-Ag
HeLa target
Primary T effector
Activator
Blocker
